# Comment on “Multidimensional kinetic study on the organocatalyzed ring-opening polymerization (ROP) of l-lactide *via* a robotic high-throughput flow platform” by B. Zhang and T. Junkers, *Chem. Sci.*, 2026, **17**, 4706

**DOI:** 10.1039/d6sc02040b

**Published:** 2026-06-15

**Authors:** Glenn Keith Kim Clothier, Simon Harrisson

**Affiliations:** a Univ. Bordeaux, CNRS, Bordeaux INP, LCPO UMR 5629 Pessac F-33600 France simon.harrisson@u-bordeaux.fr

## Abstract

Zhang and Junkers recently reported a multidimensional kinetic study of the ring-opening polymerization of l-lactide (LLA) catalyzed by 1,5,7-triazabicyclo[4.4.0]dec-5-ene (TBD) and initiated by 4-methylbenzyl alcohol (MBA). Reanalysis of their conversion–time data using an initial-rate approach suggests that the kinetics are consistent with an overall rate dependence close to *R*_p_ ∝ [LLA]^1^[TBD]^0.5^[MBA]^0.5^. The time-series data also indicate that the reaction rate decreases as the polymerization proceeds, suggesting the occurrence of catalyst deactivation under some conditions.

## Abstract 2

Reanalysis of flow polymerization kinetics of TBD-catalyzed l-lactide polymerization using an initial-rate approach reveals deviations from first-order behavior and supports a revised rate law.

## Graphical abstract

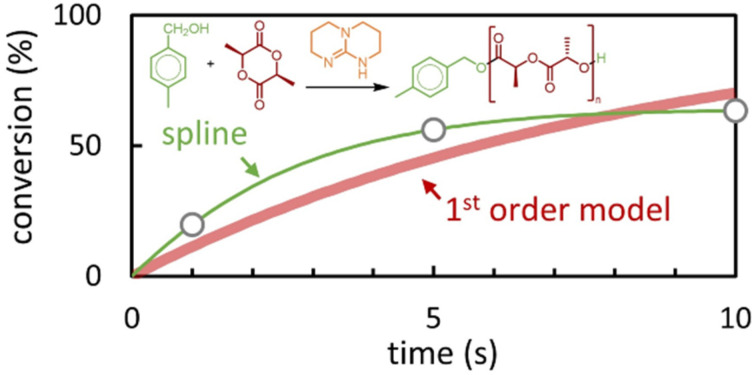

## Introduction

**Scheme 1:**
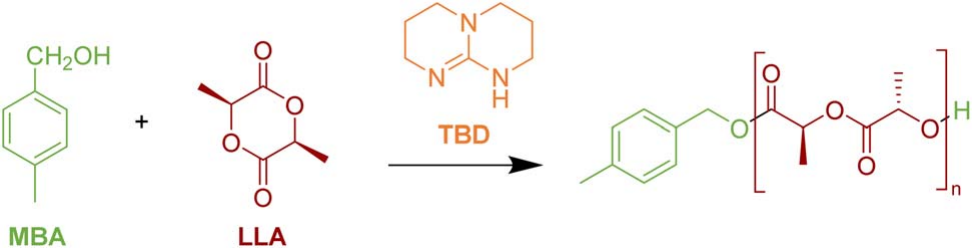
TBD-catalyzed polymerization of l-lactide (LLA), initiated by 4-methylbenzyl alcohol (MBA).

In a recent paper,^1^ Zhang and Junkers presented the results of a multidimensional kinetic study of the ring-opening polymerization (ROP) of l-lactide (LLA), catalyzed by 1,5,7-triazabicyclo[4.4.0]dec-5-ene (TBD) and initiated by 4-methylbenzyl alcohol (MBA) (Scheme 1). Their study generated conversion data for polymerizations carried out in continuous flow with residence times of 1, 5 and 10 s. A wide range of monomer, initiator, and catalyst concentrations were investigated in the course of three series of experiments.

First, a monomer concentration sweep varied the monomer concentration from 0.2 to 0.8 M while maintaining a constant mole ratio of LLA : MBA : TBD of 100 : 1 : 0.5. Second, a monomer-to-catalyst ratio series fixed the LLA : MBA ratio at 100 : 1 while varying the LLA : TBD ratio from 200 to 1200. Third, a degree of polymerization (DP) series fixed the LLA : TBD ratio at 300 : 1 while varying the LLA : MBA ratio from 50 to 150.

The monomer concentration sweep experiments were performed at 35, 30, 20, 10 and 0 °C. The monomer-to-catalyst and DP series were carried out at 20 °C with lactide concentrations of 0.2, 0.35, 0.5 and 0.7 M. Conversion was determined immediately after quenching the reaction, with a reported absolute measurement error of <5%.

The resulting dataset represents one of the most extensive kinetic studies of lactide ROP reported to date, and provides valuable insight into this important polymerization. As the authors note, despite substantial interest in organocatalyzed lactide polymerization, relatively few detailed kinetic studies of this^2,3^ and closely related^4–6^ systems have been reported.

To analyze this dataset, Zhang and Junkers proposed a kinetic analysis in which they first fitted a 3D surface to the data using MATLAB. This surface was then sliced into time series consisting of 10 conversion data points at 1 s intervals from 1 to 10 s, each slice corresponding to a single set of monomer, initiator and catalyst concentrations. The conversion data were then transformed to ln([LLA]_0_/[LLA]_*t*_) and fitted to a straight line that was constrained to pass through the origin. The slope of this line, *k*_obs_, was taken to be the apparent rate constant for the polymerization, and formed the basis of subsequent kinetic analysis.

**Fig. 1:**
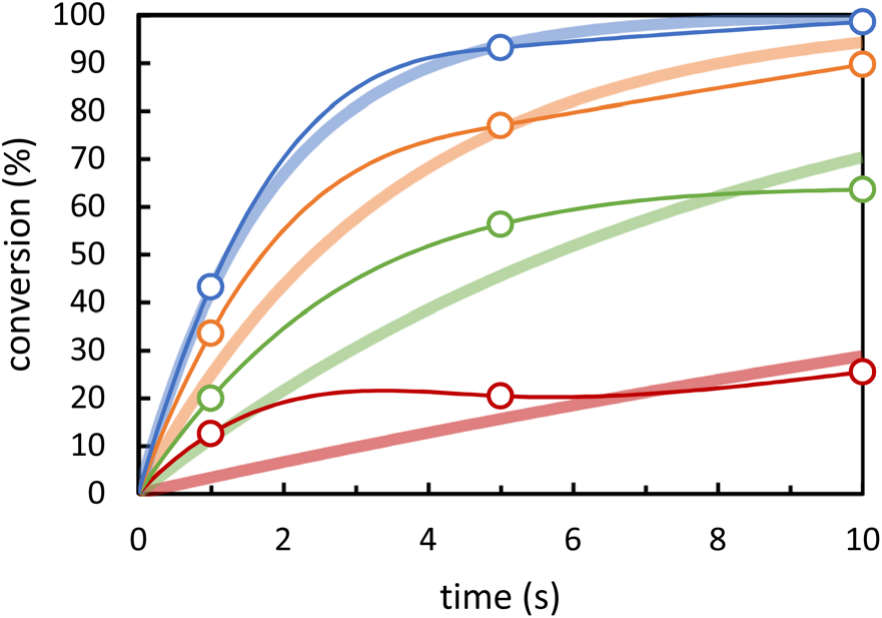
Conversion data from monomer concentration sweep experiments at 35 °C, [LLA]_0_ = 0.2 M (red), 0.4 M (green), 0.6 M (orange) and 0.8 M (blue), [LLA] : [MBA] : [TBD] = 100 : 1 : 0.5. Broad lines show best first-order model fit (conversion = 1 − exp(−*k*_obs_·*t*)). Narrow lines show cubic spline fit (details in SI).

This approach is widely used in polymerization kinetics, and corresponds to the expected behavior of a polymerization that is first-order in monomer with a constant concentration of propagating species. Fig. 1 shows the conversion data taken from monomer concentration sweep experiments at 35 °C over a range of monomer concentrations, at residence times of 1, 5 and 10 s. The broad lines correspond to the best fit obtained using a first-order kinetic model. Closer inspection of the underlying conversion–time data suggests, however, that the reaction kinetics may not be fully captured by this model.

In three of the four cases shown, the model underestimates the conversion at the earliest time point by more than 10% (absolute), more than twice the expected measurement uncertainty. The data therefore appear to indicate that the reaction proceeds with a relatively rapid initial rate that declines more sharply than predicted by the first-order model as conversion increases, and in some cases appears to plateau well below full conversion. Such behavior may arise from several factors, including a decrease in the concentration of active propagating species, depolymerization processes that impose a limiting conversion, or an intrinsic deviation from first-order dependence on monomer concentration.

We therefore considered whether a more direct estimate of the initial polymerization rate could be obtained by (1) fitting a smooth curve to the experimental data, constrained to pass through the origin; and (2) evaluating the derivative of this curve at *t* = 0 to obtain the initial rate of change in LLA concentration, (d[LLA]/d*t*)_0_. The narrow lines in Fig. 1 illustrate this approach. The curves are cubic splines that are continuous in their first and second derivatives, pass through the point (0, 0), and are set to have a second derivative of zero at *t* = 10 s.

As shown by the curve at [LLA]_0_ = 0.8 M, when the experimental data are well described by the first-order model, the spline closely follows the model during the first second of the reaction. When the data deviate from the first-order model, however, the spline fit yields a substantially higher estimate of the initial rate.

**Fig. 2:**
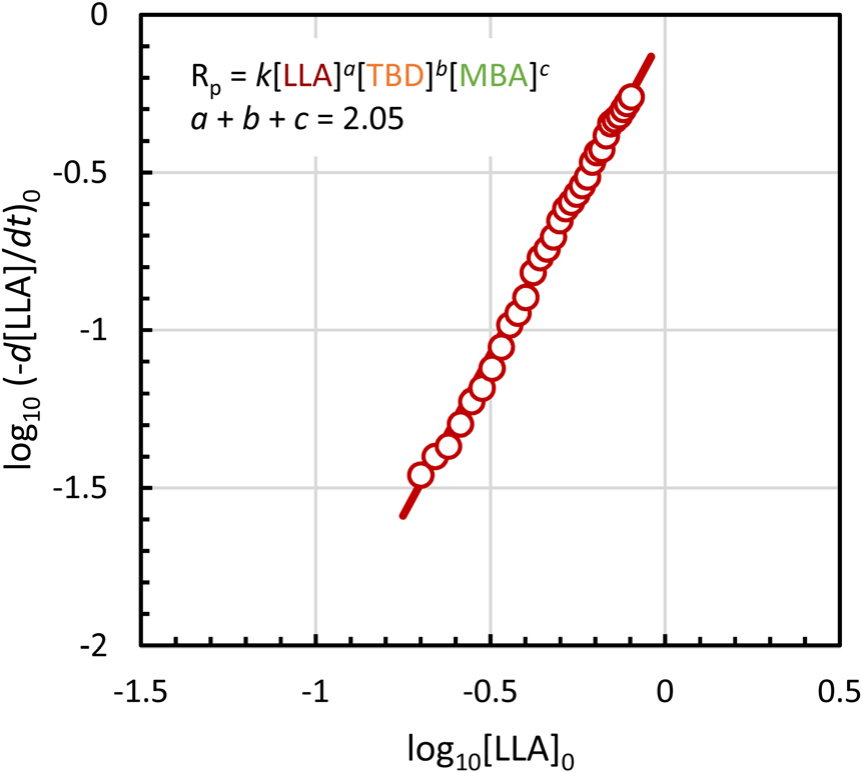
Log–log plot of initial rate of LLA consumption *vs.* initial LLA concentration for monomer concentration sweep experiments at 20 °C (Table S3). [LLA] : [MBA] : [TBD] = 100 : 1 : 0.5.

Applying this analysis to the dataset obtained from the monomer concentration sweep experiments at 20 °C yields a log–log relationship between the initial reaction rate and the initial monomer concentration with an apparent slope of approximately 2 (Fig. 2). This value corresponds to the combined reaction orders with respect to monomer, catalyst and initiator. Similar overall reaction orders are observed at 30 °C and 35 °C, while lower apparent reaction orders are obtained at 10 °C and 0 °C, likely due to limitations in time resolution for these very fast reactions (Fig. S1).

**Fig. 3:**
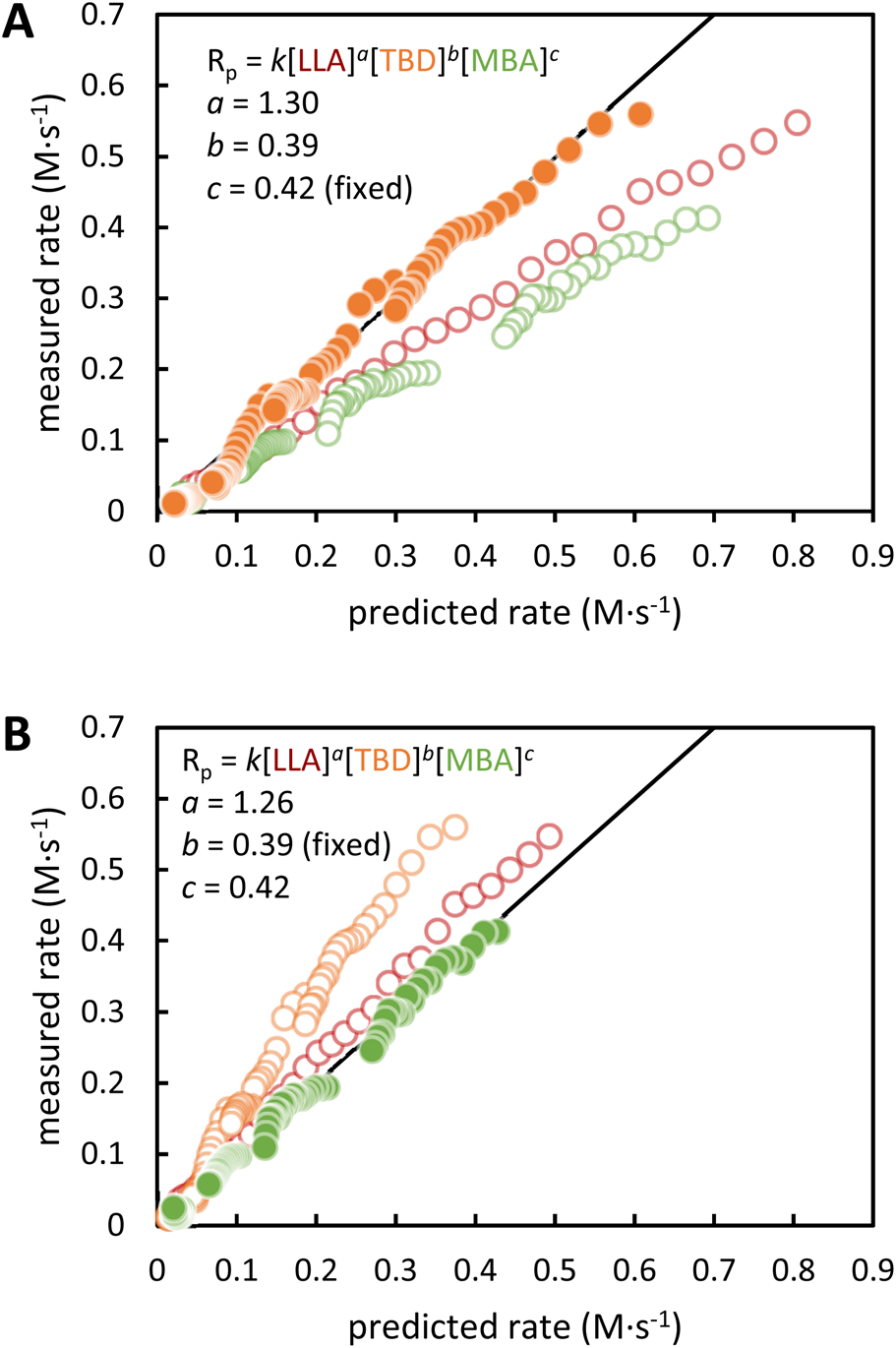
Comparison of predicted and measured initial rates (−d[LLA]/d*t*)_0_ for monomer-to-catalyst ratio sweep (A) and degree of polymerization sweep (B) experiments. In each graph, the model is fit to the filled points, while the open circles represent the predictions of the model applied to the remaining experiments. In each panel, the exponent not determined by the corresponding dataset is obtained from the complementary dataset. Red: monomer concentration sweep (Tables S3–S7); orange: monomer-to-catalyst ratio sweep (Tables S8–S10); green: DP sweep (Tables S11–S15).

The monomer-to-catalyst ratio sweep experiments allow independent determination of the order of reaction in catalyst and the sum of the orders in monomer and initiator; while the DP sweep experiments allow the order of reaction in initiator to be determined separately from the sum of the orders in monomer and catalyst. Reaction orders were determined by weighted multiple linear regression of ln(−d[LLA]/d*t*)_0_ on ln[TBD]_0_ and ln[LLA]_0_, or ln[MBA]_0_ and ln[LLA]_0_. The corresponding parity plots for predicted and measured initial rates are shown in Fig. 3A and B. As each dataset only constrains two of the three exponents, one exponent must be fixed to allow comparison of all points. Thus in Fig. 3A, the order in MBA is fixed to the value obtained in Fig. 3B, while in Fig. 3B, the order in TBD is fixed to the value obtained in Fig. 3A. This analysis suggests a reaction order of 0.39 with respect to catalyst, and 0.42 with respect to initiator.

**Fig. 4:**
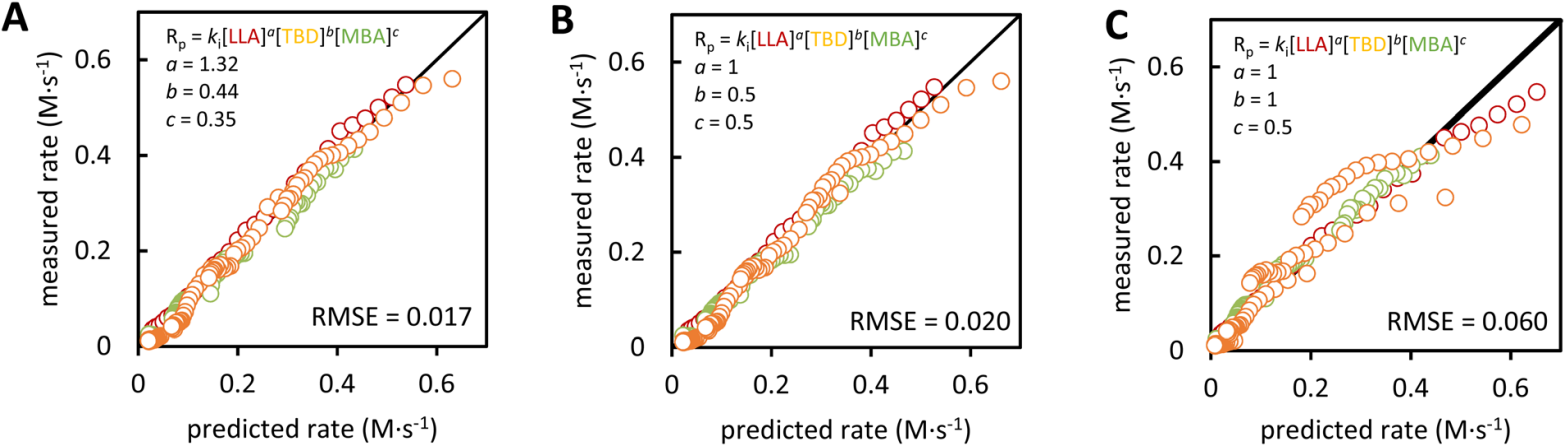
Comparison of predicted and measured initial rates (−d[LLA]/d*t*)_0_ for all experiments, allowing *k*_*i*_ to vary depending on the experiment series (*k*_1_: monomer-to-catalyst ratio sweep; *k*_2_: monomer concentration and DP sweep). (A) Empirical best fit from multiple weighted linear regression; (B) first-order in [LLA], half-order in [TBD] and [MBA]; (C) first-order in [LLA] and [TBD], half-order in [MBA]. Red: monomer concentration sweep (Tables S3–S7); orange: monomer-to-catalyst ratio sweep (Tables S8–S10); green: DP sweep (Tables S11–S15).

It can also be observed from Fig. 3 that while the rates of the DP sweep experiments are consistent with those of the monomer concentration sweep experiments, the catalyst sweep experiments display faster rates. It was not possible to obtain a good fit to all data simultaneously (Fig. S2). However, all data could be fit to the model given in eqn (1):1
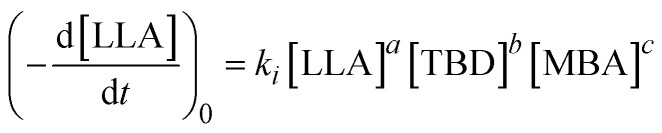
if *k*_*i*_ was allowed to take one value (*k*_1_) for the monomer-to-catalyst series of experiments, and a different value (*k*_2_) for the remaining experiments. In this case, reaction orders of 1.32 in monomer, 0.44 in catalyst and 0.35 in initiator were obtained (Fig. 4A).

This empirical model predicts the initial rate of the polymerization with a root mean square error (RMSE) of 0.017. Equally good agreement between predicted and measured rates is obtained by setting the order in monomer to 1 and the order in both catalyst and initiator to 0.5 (RMSE = 0.020, Fig. 4B). However, forcing the order in monomer and catalyst to 1 and the order in initiator to 0.5 leads to a significant reduction in the quality of the fit (RMSE = 0.060, Fig. 4C).

## Mechanistic interpretation

A reaction order of 1 in monomer and 0.5 in both catalyst and initiator would be consistent with a mechanism in which a small fraction of MBA is deprotonated by TBD. In this case, the concentration of 4-methylbenzyloxide (MBA^−^) will be governed by the acid–base equilibrium (eqn (2)).2
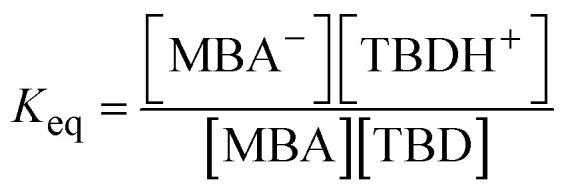
Assuming that the extent of deprotonation is negligible compared to the concentration of the initiator and catalyst,††While the p*K*_a_ of MBA has not yet been reported in an aprotic solvent, a low degree of deprotonation appears likely given the large difference in p*K*_a_ values in DMSO between TBDH^+^ (15.3) and aliphatic alcohols such as MeOH (29.0) or EtOH (29.8).^15,16^ A similar difference in p*K*_a_ would be expected in other aprotic solvents.^17^ the concentration of the deprotonated alcohol will be given by eqn (3):3
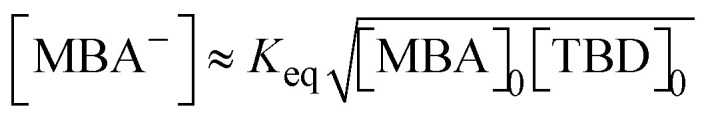
The overall rate equation is then given by eqn (4):4
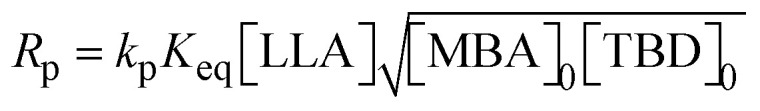
The apparent deviations from first-order kinetics in the time-series data of Fig. 1 may arise as a result of a reversible propagation step,^7^ or from side reactions that lead to degradation of the catalyst. While a kinetic model that incorporates reversible propagation provides a good empirical fit to the kinetics (Fig. S3), the fitted values of the equilibrium monomer constant ([LLA]_eq_) vary substantially with the initial monomer concentration, ranging from 0.004 to 0.17 M at 35 °C (Fig. S4 and Table S16). This is inconsistent with the assumptions of the model, as [LLA]_eq_ should be independent of the initial monomer concentration. Additionally, literature values for [LLA]_eq_ under comparable conditions are generally low (0.001–0.033 M at 20 °C, depending on catalyst and solvent (Table S17),^7–9^ and many of the reactions in the dataset proceed to near-complete conversion. This suggests that neglecting depolymerization will not introduce significant error to the kinetic analysis.

Following a reviewer's suggestion, we tested this assumption using values for [LLA]_eq_ calculated from data obtained under similar conditions^9^ (1,8-diazabicyclo[5.4.0]undec-7-ene-catalyzed ROP of LLA in dioxane, Table S18). Correcting the initial rate to account for the contribution of depolymerization led to a small decrease in the apparent overall reaction order (from 2.05 to 1.99 at 20 °C, Fig. S5), which was more pronounced at higher temperatures. A corresponding decrease was observed in the apparent reaction order with respect to [LLA] (Fig. S6 and S7A), while the dependence on [TBD] and [MBA] was unaffected. The data continued to be well described by the simplified rate law *R*_p_ = *k*_*i*_[M]^1^[TBD]^0.5^[MBA]^0.5^ (Fig. S7B), and poorly fitted by the rate law *R*_p_ = *k*_*i*_[M]^1^[TBD]^0.5^[MBA]^1^ (Fig. S7C).

**Scheme 2:**
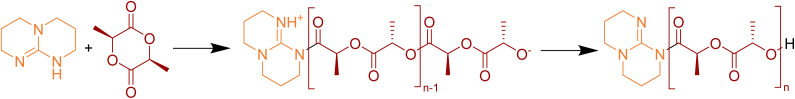
A possible mechanism of catalyst degradation in TBD-catalyzed polymerization of l-lactide, involving direct initiation of polymerization by TBD followed by deprotonation of the zwitterionic adduct to form an *N*-acyl guanidine species with reduced catalytic activity.^14^

This leaves deactivation of the catalyst during the polymerization as the likely cause of most of the observed retardation. In particular, formation of a zwitterionic adduct between TBD and lactide,^10,11^ followed by propagation (Scheme 2), could lead to an effective reaction order in lactide that is greater than 1, since lactide would participate in both the initiation and propagation steps. Further experiments would be required, however, to determine the order of reaction with respect to monomer with greater precision.

The proposed pathway could also lead to a gradual loss of catalyst activity as a result of deprotonation of the TBD adduct, leading to irreversible formation of a less-active *N*-acyl guanidine species. Several studies have demonstrated that TBD can initiate polymerization of lactide in the absence of added alcohol initiator,^10,12–14^ and a stable *N*-acyl guanidine species has been observed in the resulting polymer using MALDI mass spectrometry.^14^ While we cannot definitively state that this product is formed under the present conditions, we believe it represents a plausible mechanism for catalyst deactivation.

## Conclusions

Reanalysis of the kinetic data presented by Zhang and Junkers from an initial rate perspective suggests that the TBD-catalysed polymerization of l-lactide is best described by a rate equation of the form *R*_p_ = *k*[M]^1.32^[TBD]^0.44^[MBA]^0.35^. A simpler rate law, *R*_p_ = *k*[M]^1^[TBD]^0.5^[MBA]^0.5^, provides comparable goodness of fit, and cannot be excluded on the basis of the present dataset. The latter rate equation is consistent with the mechanism proposed by Zhang and Junkers, in which the rate determining step is ring-opening of the lactide by a deprotonated alcohol, produced in low concentration by reaction between TBD and MBA. Under most of the conditions investigated, the rate of polymerization slows as the reaction proceeds, suggesting the occurrence of side-reactions that gradually deactivate the catalyst. The dataset reported by Zhang and Junkers therefore provides a valuable foundation for further mechanistic analysis of this important polymerization system.

## Author contributions

Glenn K. K. Clothier: methodology, writing – review & editing. Simon Harrisson: conceptualization, methodology, writing – original draft, writing – review & editing.

## Conflicts of interest

There are no conflicts to declare.

## Supplementary Material

SC-OLF-D6SC02040B-s001

SC-OLF-D6SC02040B-s002

## Data Availability

No primary research results, software or code have been included and no new data were generated as part of this comment. Supplementary information (SI): full details of model fitting and regression analysis (Tables S3–S15, Excel worksheet containing all calculations). See DOI: https://doi.org/10.1039/d6sc02040b.
