# Supplementary material for: Comment on “Multidimensional kinetic study on the organocatalyzed ring-opening polymerization (ROP) of l-lactide via a robotic high-throughput flow platform” by B. Zhang and T. Junkers, Chem. Sci., 2026, 17, 4706
Source: Chem Sci. 2026 Jun 15;17(24):12142–6. doi: 10.1039/d6sc02040b (PMC13267183; doi:10.1039/d6sc02040b)
Supplement: SC-017-D6SC02040B-s002 [file SC-017-D6SC02040B-s002.pdf]

Supplementary Information (SI) for Chemical Science.
This journal is © The Royal Society of Chemistry 2026

Comment on "Multidimensional kinetic study on the organocatalyzed ring-opening polymerization (ROP) of l-lactide via a robotic high-throughput flow platform"by B. Zhang and T. Junkers, *Chem. Sci.*, 2026, 17, 4706-4714

Glenn Keith Kim Clothier and Simon Harrisson*

Univ. Bordeaux, CNRS, Bordeaux INP, LCPO, UMR 5629, Pessac, F-33600 France

Email: simon.harrisson@u-bordeaux.fr

Cubic spline fitting

Cubic spline interpolators were generated from data points at 1 s, 5 s and 10 s (obtained from Tables S6-S14 and S17-S20 of Zhang and Junkers, *Chem. Sci.* 2026, **17**, 4706-4714). The curves consisted of two cubic sections and were constrained to have continuous first and second derivatives, pass through the point (0,0) corresponding to zero conversion (*X*) at t = 0, and have a second derivative of zero at t = 10 s.

The general formula for the spline interpolator is:

$0 \le t \le 5$: $X = f_1(t) = a_1t^3 + a_2t^2 + a_3t \ \#(S1a)$

$5 \le t \le 10$: $X = f_2(t) = \ a_4t^3 + a_5t^2 + a_6t + a_7 \ \#(S1b)$

This leads to a system of linear equations for the coefficients:

$$X_1 = f_1(1) = a_1 + a_2 + a_3 \ \#(S2a)$$
$$X_5 = f_1(5) = 125a_1 + 25a_2 + 5a_3 \ \#(S2b)$$
$$X_5 = f_2(5) = 125a_4 + 25a_5 + 5a_6 + a_7 \ \#(S2c)$$
$$X_{10} = f_2(10) = 1000a_4 + 100a_5 + 10a_6 + a_7 \ \#(S2d)$$
$$f_1^{'}(5) - f_2^{'}(5) = 75a_1 + 10a_2 + a_3 - 75a_4 - 10a_5 - a_6 = 0 \ \#(S2e)$$
$$f_1^{''}(5) - f_2^{''}(5) \ = 30a_1 + 2a_2 - 30a_4 - 2a_5 = 0 \ \#(S2f)$$
$$f_2^{''}(10) = 60a_4 + 2a_5 = 0 \ \ \#(S2g)$$

Solving this system of equations gives each coefficient, a_i, as a linear combination of the experimental conversion values X_1, X_5, and X_{10}, measured at t = 1, 5 and 10 s:

$$a_i = c_{i1}X_1 + c_{i5}X_5 + c_{i10}X_{10} \ \#(S3)$$

The coefficients c_{i1}, c_{i5}, c_{i10} for calculation of a_1-a_8 are given in Table S1.

Table S1: Coefficients for calculation of cubic spline interpolator of conversions

a_i[a]	c_{i1}	c_{i5}	c_{i10}
a_1	1/24	-49/3000	1/250
a_2	-1/2	37/250	-3/125
a_3	35/24	-79/600	1/50
a_4	-1/120	97/15000	-3/1250
a_5	1/4	-97/500	9/125
a_6	-55/24	947/600	-23/50
a_7	25/4	-57/20	4/5

[a] $a_i = c_{i1}.X_1 + c_{i5}.X_5 + c_{i10}.X_{10}$; $X = a_1t^3 + a_2t^2 + a_3t$, $(0 \le t \le 5)$; $X = a_4t^3 + a_5t^2 + a_6t + a_7$, $(5 \le t \le 10)$.

The first derivative of the spline interpolator at t = 0 is simply the coefficient a_3, equal to 35 X_1/24 -79 X_5/600 + X_{10}/50. Multiplying this value by the initial lactide concentration, $[LLA]_0$, gives the initial rate of monomer consumption, $(d[LLA]/dt)_0$.

Regression analysis:

1. Determination of k_{obs} (Figure 1, Tables S2-S14)

The apparent first-order rate constant, k_{obs}, was determined by weighted linear regression of the transformed conversion-time data (X_1, X_5, X_{10}, Tables S2-S14). Conversion data, X_i, were transformed to ξ_i, where $\xi_i = -\ln(1-X_i)$. When X_i = 100%, it was set to 99.99% in order to allow calculation of k_{obs}. A weight matrix **W** was defined as $W_{ii} = (1-X_i)^2$, $W_{ij} = 0$ ($i \neq j$). The purpose of the weight matrix is to correct for distortions in the error structure introduced by the logarithmic transformation. The apparent rate constant was then calculated from equation:

$$k_{obs} = \frac{\sum_i t_i W_{ii} \xi_i}{\sum_i t_i W_{ii} t_i} \#(S4)$$

2. Determination of overall reaction order (Figure 2, Figure S2)

The overall reaction order was determined by weighted linear regression of the logarithm of the initial rate ($(-d[LLA]/dt)_0$, Tables S3-S7) on the initial monomer concentration $[LLA]_0$ for the monomer concentration sweep data. A weight matrix **W** was defined as $W_{ii} = (d[LLA]/dt)_0^2$, $W_{ij} = 0$ ($i \neq j$). The purpose of the weight matrix is to correct for distortions in the error structure introduced by the logarithmic transformation.

$$\begin{bmatrix} \log_{10} k \\ a \end{bmatrix} = (X^T W X)^{-1} X^T W Y \#(S5)$$

Where **X** is the information matrix whose entries are $X_{i1} = 1$, $X_{i2} = ([LLA]_0)_i$, and **Y** is the vector of initial rates $Y_i = (\log_{10}(-d[LLA]/dt)_0)_i$.

The results of this analysis are shown in Table S2.

Table S2. Regression to determine overall reaction order

T (°C)	$\log_{10} k$	a	RMSE[a]
35	-0.196	1.98	0.011
30	-0.083	1.91	0.010
20	-0.051	2.05	0.009
10	0.002	1.57	0.018
0	-0.015	1.33	0.017

[a] RMSE is the root mean square error of the model, calculated as $RMSE = \sqrt{\frac{\sum_i \left(-\left(\frac{d[LLA]}{dt}\right)_{0i} - k[LLA]_{0i}^{\ a}\right)^2}{N-2}}$. The number of data points, N, is 31.

3. Determination of reaction order in catalyst (Figure 3A) and initiator (Figure 3B)

The reaction order in catalyst was determined by multiple weighted linear regression of the logarithm of the initial rate ($(-d[LLA]/dt)_0$, Tables S8-S11) on the initial monomer concentration $[LLA]_0$ and the catalyst concentration for the catalyst-to-monomer ratio sweep data. A weight matrix **W** was defined as $W_{ii} = (d[LLA]/dt)_0^2$, $W_{ij} = 0$ ($i \neq j$). The purpose of the weight matrix is to correct for distortions in the error structure introduced by the logarithmic transformation.

The parameters of the model $\ln R_p = \ln(k) + a\ln[LLA]_0 + b\ln[TBD]_0$ were determined from equation S6.

$$\begin{bmatrix} \ln(k) \\ a \\ b \end{bmatrix} = \left(X^T W X\right)^{-1} X^T W Y \#(S6)$$

Where **X** is the information matrix whose entries are $X_{i1} = 1$, $X_{i2} = ([LLA]_0)_i$, $X_{i3} = ([TBD]_0)_i$, and **Y** is the vector of initial rates $Y_i = (\ln(-d[LLA]/dt)_0)_i$.

The best fit to the data was obtained with the following model (Figure 3A):

$\left(-\frac{d[\hat{L}LA]}{dt}\right)_0 =$ $10.4 \cdot [LLA]_0^{1.72}[TBD]_0^{0.39}$ (84 data points, 3 parameters, RMSE = 0.009)

A similar method was applied to the degree of polymerization sweep data (Tables S12-S15) to obtain the model (Figure 3B):

$\left(-\frac{d[\hat{L}LA]}{dt}\right)_0 =$ $4.56 \cdot [LLA]_0^{1.65}[MBA]_0^{0.42}$ (84 data points, 3 parameters, RMSE = 0.019)

In both cases, the RMSE was determined from the sum of squared residuals between the initial rate of reaction obtained from the spline fit, and the predicted rate of reaction, $\left(-\frac{d[\hat{L}LA]}{dt}\right)_0$, divided by N-p, where N is the number of data points and p the number of parameters.

4. Determination of reaction order in monomer, catalyst and initiator (Figure 4A, Figure S2)

Weighted linear regression of the logarithm of the initial rate ($(-d[LLA]/dt)_0$, Tables S3-S15) on the initial monomer concentration $[LLA]_0$, catalyst concentration $[TBD]_0$ and initiator concentration $[MBA]_0$ was carried out on all data. A weight matrix **W** was defined as $W_{ii} = (d[LLA]/dt)_0^2$, $W_{ij} = 0$ ($i \neq j$). The purpose of the weight matrix is to correct for distortions in the error structure introduced by the logarithmic transformation.

The parameters of the model ln R_p = ln(k) + $a$ln[LLA]$_0$ + $b$ln[TBD]$_0$ + $c$ln[MBA]$_0$ were determined from equation S7:

$$\begin{bmatrix} \ln(k) \\ a \\ b \\ c \end{bmatrix} = \left(X^T W X\right)^{-1} X^T W Y \#(S7)$$

Where **X** is the information matrix whose entries are X_{i1} = 1, X_{i2} = ([LLA]$_0$)$_i$, X_{i3} = ([TBD]$_0$)$_i$, X_{i4} = ([MBA]$_0$)$_i$, and **Y** is the vector of initial rates Y_i = (ln(-d[LLA]/dt)$_0$)$_i$.

The best fit to the data was obtained with the following model (Figure S2):

$\left(-\frac{d[\hat{L}LA]}{dt}\right)_0 =$ 4.59·[LLA]$_0^{1.74}$[TBD]$_0^{0.17}$[MBA]$_0^{0.16}$ (199 data points, 4 parameters, RMSE = 0.041)

A significant improvement in the fit was obtained by allowing k to vary depending on the reaction series. This led to the following model:

$\left(-\frac{d[\hat{L}LA]}{dt}\right)_0 =$ k_i·[LLA]$_0^{1.32}$[TBD]$_0^{0.44}$[MBA]$_0^{0.35}$ (199 data points, 5 parameters, RMSE = 0.017)

The values of k_i are k_1 (monomer concentration sweep and degree of polymerization sweep) : 44.6; k_2 (catalyst-to-monomer ratio sweep) : 69.3.

In both cases, the RMSE was determined from the sum of squared residuals between the initial rate of reaction obtained from the spline fit, and the predicted rate of reaction, $\left(-\frac{d[\hat{L}LA]}{dt}\right)_0$, divided by N-p, where N is the number of data points and p the number of parameters.

5. Fixed reaction order in monomer, catalyst and initiator (Figure 4B, Figure 4C)

The data were also fit to a model in which the order in monomer was fixed to 1, and the order in catalyst and initiator were fixed to 0.5. Again, k was allowed to vary depending on the reaction series. The following model was obtained:

$\left(-\frac{d[\hat{L}LA]}{dt}\right)_0 =$ k_i·[LLA]$_0$[TBD]$_0^{0.5}$[MBA]$_0^{0.5}$ (199 data points, 2 parameters, RMSE = 0.020)

The values of k_i are k_1 (monomer concentration sweep and degree of polymerization sweep) : 116 M^{-1}·s^{-1}; k_2 (catalyst-to-monomer ratio sweep) : 191 M^{-1}·s^{-1}.

Finally, the model proposed by Zhang and Junkers[1] was fit to the data: in this model the order in monomer and catalyst is set to 1, and the order in initiator is set to 0.5. Again, k was allowed to vary depending on the reaction series. The following model was obtained:

$\left(-\frac{d[\hat{L}LA]}{dt}\right)_0 =$ k_i·[LLA]$_0$[TBD]$_0$[MBA]$_0^{0.5}$ (199 data points, 2 parameters, RMSE = 0.060)

The values of k_i are k_1 (monomer concentration sweep and degree of polymerization sweep) : 2279 $M^{-1.5}\cdot s^{-1}$; k_2 (catalyst-to-monomer ratio sweep) : 5312 $M^{-1.5}\cdot s^{-1}$.

In both cases, the RMSE was determined from the sum of squared residuals between the initial rate of reaction obtained from the spline fit, and the predicted rate of reaction, $\left(-\frac{d[\hat{L}LA]}{dt}\right)_0$, divided by N-p, where N is the number of data points and p the number of parameters.

6. First-order model incorporating a reversible propagation step (Figure S3, Figure S4)

The kinetics of a polymerization with a reversible propagation step in which the forward reaction is first order in monomer are described by equation S8:

$$R_p = -\frac{d[M]}{dt} = k_p[M][P^*] - k_{dp}[P^*] \#(S8)$$

In this equation, M represents the monomer and P* the propagating species.

Integrating this equation and setting $[LLA]_{eq} = k_{dp}/k_p$ and $k_{obs} = k_p[P^*]$ gives equation S9, relating conversion, X, to time, *t* :

$$X = \left(1 - \frac{[LLA]_{eq}}{[LLA]_0}\right)\left(1 - \exp\left(-k_{obs}t\right)\right) \#(S9)$$

The conversion data obtained from the monomer sweep experiments at 35°C (Table S3) were fit to this model using nonlinear least squares regression. Selected examples of the fit are shown in Figure S3, and the values thus obtained for $[M]_{eq}$ and k_{obs} are plotted in Figure S4, and tabulated in Table S16.

7. Correction of initial rates to compensate for depolymerization

Evaluating equation S8 at t = 0, [M] = $[LLA]_0$ gives equation S10:

$$(R_p)_0 = -\left(\frac{d[LLA]}{dt}\right)_0 = k_p[LLA]_0[P^*]_0 - k_{dp}[P^*]_0 = k_p[P^*]_0([LLA]_0 - [LLA]_{eq}) \#(S10)$$

Setting $[P^*] \sim [LLA]^{a-1}[TBD]^b[MBA]^c$, this gives the corrected rate equation S11:

$$(R_{p,corrected})_0 = -\frac{[LLA]_0}{([LLA]_0 - [LLA]_{eq})}\left(\frac{d[LLA]}{dt}\right)_0 = k_p[P^*]_0[M]_0 = k[LLA]_0^a[TBD]_0^b[MBA]_0^c \#(S11)$$

Thus the contribution of depolymerization to the observed rate can be compensated for by multiplying the rate of monomer consumption by a correction factor, $[LLA]_0/([LLA]_0 - [LLA]_{eq})$.

Equilibrium concentrations $[LLA]_{eq}$ at 0, 10, 20, 30 and 35°C were estimated from the data of Cederholm et al. (L. Cederholm, J. Wohlert, P. Olsén, M. Hakkarainen and K. Odelius, *Angew. Chem. Int. Ed.* 2022, **61**, e202204531), obtained at 55-85°C in dioxane solution using DBU as catalyst, and are given in Table S18.

Table S3. Initial rate of monomer consumption, k_{obs} and depolymerization-corrected rate of monomer consumption for monomer concentration sweep data at 35°C

$[LLA]_0$ (M)[a]	$[MBA]_0$ (mM)[a]	$[TBD]_0$ (mM)[a]	X_1 (%)[a]	X_5 (%)[a]	X_{10} (%)[a]	$\left(\frac{dX}{dt}\right)_0$ (s^{-1})[b]	$\left(\frac{-d[LLA]}{dt}\right)$ ($M \cdot s^{-1}$)[c]	k_{obs} (s^{-1})[d]	$k_{obs}[LLA]_0$ ($M \cdot s^{-1}$)[e]	*correction factor*[f]	$(R_{p,corr})_0$ ($M \cdot s^{-1}$)[g]
0.20	2.0	1.0	12.47	20.36	25.40	0.1601	0.0320	0.0340	0.0068	1.283	0.0411
0.22	2.2	1.1	14.42	25.33	28.94	0.1827	0.0402	0.0407	0.0090	1.251	0.0503
0.24	2.4	1.2	16.16	28.44	32.20	0.2047	0.0491	0.0465	0.0112	1.225	0.0602
0.26	2.6	1.3	15.56	30.77	35.82	0.1936	0.0503	0.0525	0.0136	1.204	0.0606
0.28	2.8	1.4	16.02	35.96	39.32	0.1941	0.0544	0.0602	0.0169	1.187	0.0645
0.30	3.0	1.5	18.13	41.53	42.64	0.2182	0.0655	0.0684	0.0205	1.172	0.0768
0.32	3.2	1.6	17.39	43.23	46.85	0.2061	0.0659	0.0765	0.0245	1.160	0.0765
0.34	3.4	1.7	18.37	48.06	50.95	0.2148	0.0730	0.0868	0.0295	1.149	0.0839
0.36	3.6	1.8	18.62	47.64	55.29	0.2199	0.0792	0.0957	0.0345	1.140	0.0902
0.38	3.8	1.9	18.46	49.86	60.00	0.2156	0.0819	0.1076	0.0409	1.131	0.0927
0.40	4.0	2.0	19.88	56.21	63.50	0.2286	0.0914	0.1214	0.0485	1.124	0.1028
0.42	4.2	2.1	22.33	59.65	65.62	0.2602	0.1093	0.1306	0.0548	1.117	0.1221
0.44	4.4	2.2	23.06	60.86	68.60	0.2699	0.1187	0.1411	0.0621	1.111	0.1320
0.46	4.6	2.3	22.09	63.79	72.52	0.2527	0.1162	0.1568	0.0721	1.106	0.1285
0.48	4.8	2.4	24.27	64.10	76.01	0.2847	0.1367	0.1718	0.0825	1.101	0.1505
0.50	5.0	2.5	26.17	66.66	81.01	0.3101	0.1550	0.1984	0.0992	1.097	0.1700
0.52	5.2	2.6	27.29	69.95	84.98	0.3229	0.1679	0.2259	0.1175	1.093	0.1835
0.54	5.4	2.7	28.35	71.67	86.29	0.3363	0.1816	0.2388	0.1290	1.089	0.1978
0.56	5.6	2.8	27.76	75.58	88.07	0.3229	0.1808	0.2598	0.1455	1.085	0.1963
0.58	5.8	2.9	30.05	76.82	88.76	0.3548	0.2058	0.2725	0.1581	1.082	0.2227
0.60	6.0	3.0	33.49	76.90	89.59	0.4051	0.2430	0.2855	0.1713	1.079	0.2623
0.62	6.2	3.1	34.36	79.91	91.12	0.4141	0.2567	0.3124	0.1937	1.077	0.2764
0.64	6.4	3.2	33.82	83.51	92.50	0.4018	0.2571	0.3402	0.2177	1.074	0.2761
0.66	6.6	3.3	34.62	84.64	93.38	0.4121	0.2720	0.3584	0.2365	1.072	0.2915
0.68	6.8	3.4	36.66	85.76	94.39	0.4406	0.2996	0.3856	0.2622	1.069	0.3204
0.70	7.0	3.5	39.32	87.95	95.46	0.4767	0.3337	0.4281	0.2997	1.067	0.3561
0.72	7.2	3.6	39.75	89.49	97.47	0.4814	0.3466	0.4720	0.3398	1.065	0.3692
0.74	7.4	3.7	39.59	89.99	99.49	0.4788	0.3543	0.4864	0.3599	1.063	0.3767
0.76	7.6	3.8	41.03	90.74	99.46	0.4988	0.3791	0.5083	0.3863	1.062	0.4024
0.78	7.8	3.9	42.89	92.09	98.22	0.5239	0.4086	0.5344	0.4169	1.060	0.4331
0.80	8.0	4.0	43.11	93.10	98.51	0.5258	0.4206	0.5497	0.4398	1.058	0.4452

[a] Data from Table S6, Zhang and Junkers, *Chem. Sci.* 2026, **17**, 4706-4714. [b] Derivative of cubic spline interpolator of conversion data at t = 0. [c] $\left(\frac{d[LLA]}{dt}\right)_0 = \left(\frac{dX}{dt}\right)_0 \cdot [LLA]_0$. [d] Apparent first-order rate constant obtained by fitting $\hat{X} = 1 - \exp(-k_{obs}t)$ to conversion data. [e] Initial rate of monomer consumption predicted from k_{obs}. [f] Correction factor = $[LLA]_0/([LLA]_0-[LLA]_{eq})$; $[LLA]_{eq}$ = 0.0441 M (Table S18). [g] Corrected $(R_p)_0$ to remove contribution from depolymerization: $(R_{p,corr})_0 = -d([LLA]/dt)_0 \times [LLA]_0/([LLA]_0-[LLA]_{eq})$.

Table S4. Initial rate of monomer consumption, k_{obs} and depolymerization-corrected rate of monomer consumption for monomer concentration sweep data at 30°C

$[LLA]_0$ (M)[a]	$[MBA]_0$ (mM)[a]	$[TBD]_0$ (mM)[a]	X_1 (%)[a]	X_5 (%)[a]	X_{10} (%)[a]	$\left(\frac{dX}{dt}\right)_0$ (s^{-1})[b]	$\left(\frac{-d[LLA]}{dt}\right)$ ($M\cdot s^{-1}$)[c]	k_{obs} (s^{-1})[d]	$k_{obs}[LLA]_0$ ($M\cdot s^{-1}$)[e]	*correction factor*[f]	$(R_{p,corr})_0$ ($M\cdot s^{-1}$)[g]
0.20	2.0	1.0	12.40	31.04	41.73	0.1483	0.0297	0.0605	0.0121	1.250	0.0371
0.22	2.2	1.1	15.07	31.89	43.56	0.1865	0.0410	0.0641	0.0141	1.222	0.0501
0.24	2.4	1.2	16.64	32.82	45.72	0.2086	0.0501	0.0682	0.0164	1.200	0.0601
0.26	2.6	1.3	16.93	34.98	49.38	0.2107	0.0548	0.0754	0.0196	1.182	0.0647
0.28	2.8	1.4	18.10	37.27	52.49	0.2254	0.0631	0.0826	0.0231	1.167	0.0736
0.30	3.0	1.5	21.12	38.57	55.76	0.2684	0.0805	0.0899	0.0270	1.154	0.0929
0.32	3.2	1.6	23.78	39.95	62.45	0.3067	0.0981	0.1037	0.0332	1.143	0.1122
0.34	3.4	1.7	25.22	41.27	67.28	0.3269	0.1111	0.1145	0.0389	1.133	0.1260
0.36	3.6	1.8	27.21	42.75	70.75	0.3547	0.1277	0.1235	0.0445	1.125	0.1436
0.38	3.8	1.9	28.66	49.06	74.89	0.3683	0.1400	0.1442	0.0548	1.118	0.1564
0.40	4.0	2.0	29.91	56.16	77.78	0.3778	0.1511	0.1671	0.0668	1.111	0.1679
0.42	4.2	2.1	31.45	60.34	78.51	0.3949	0.1659	0.1790	0.0752	1.105	0.1833
0.44	4.4	2.2	32.14	64.80	79.79	0.3993	0.1757	0.1935	0.0851	1.100	0.1933
0.46	4.6	2.3	33.06	65.86	80.76	0.4116	0.1893	0.2005	0.0922	1.095	0.2074
0.48	4.8	2.4	34.03	67.08	80.70	0.4241	0.2036	0.2032	0.0975	1.091	0.2221
0.50	5.0	2.5	34.82	68.86	82.70	0.4337	0.2168	0.2178	0.1089	1.087	0.2357
0.52	5.2	2.6	36.56	70.65	84.01	0.4569	0.2376	0.2308	0.1200	1.083	0.2574
0.54	5.4	2.7	38.73	71.99	83.72	0.4868	0.2629	0.2334	0.1261	1.080	0.2839
0.56	5.6	2.8	39.60	72.50	86.31	0.4993	0.2796	0.2530	0.1417	1.077	0.3011
0.58	5.8	2.9	41.03	74.05	87.35	0.5183	0.3006	0.2668	0.1547	1.074	0.3229
0.60	6.0	3.0	42.32	74.89	86.52	0.5359	0.3215	0.2638	0.1583	1.071	0.3445
0.62	6.2	3.1	43.81	76.25	84.67	0.5554	0.3444	0.2523	0.1564	1.069	0.3681
0.64	6.4	3.2	44.19	77.35	84.43	0.5595	0.3581	0.2522	0.1614	1.067	0.3819
0.66	6.6	3.3	44.49	78.67	86.52	0.5625	0.3713	0.2749	0.1815	1.065	0.3952
0.68	6.8	3.4	47.23	82.42	87.95	0.5978	0.4065	0.3012	0.2048	1.063	0.4319
0.70	7.0	3.5	47.80	84.16	88.07	0.6039	0.4227	0.3060	0.2142	1.061	0.4483
0.72	7.2	3.6	48.18	85.08	88.69	0.6083	0.4380	0.3170	0.2283	1.059	0.4638
0.74	7.4	3.7	49.60	88.59	89.55	0.6246	0.4622	0.3362	0.2488	1.057	0.4886
0.76	7.6	3.8	50.37	92.21	90.86	0.6313	0.4798	0.3647	0.2772	1.056	0.5065
0.78	7.8	3.9	50.11	92.84	93.30	0.6272	0.4892	0.4383	0.3419	1.054	0.5157
0.80	8.0	4.0	50.61	92.32	96.35	0.6358	0.5086	0.5564	0.4451	1.053	0.5354

[a] Data from Table S7, Zhang and Junkers, *Chem. Sci.* 2026, **17**, 4706-4714. [b] Derivative of cubic spline interpolator of conversion data at t = 0. [c] $\left(\frac{d[LLA]}{dt}\right)_0 = \left(\frac{dX}{dt}\right)_0 \cdot [LLA]_0$. [d] Apparent first-order rate constant obtained by fitting $\hat{X} = 1 - \exp(-k_{obs}t)$ to conversion data. [e] Initial rate of monomer consumption predicted from k_{obs}. [f] Correction factor = $[LLA]_0/([LLA]_0-[LLA]_{eq})$; $[LLA]_{eq}$ = 0.0400 M (Table S18). [g] Corrected $(R_p)_0$ to remove contribution from depolymerization: $(R_{p,corr})_0 = -(d[LLA]/dt)_0 \times [LLA]_0/([LLA]_0-[LLA]_{eq})$.

Table S5. Initial rate of monomer consumption, k_{obs} and depolymerization-corrected rate of monomer consumption for monomer concentration sweep data at 20°C

$[LLA]_0$ (M)[a]	$[MBA]_0$ (mM)[a]	$[TBD]_0$ (mM)[a]	X_1 (%)[a]	X_5 (%)[a]	X_{10} (%)[a]	$\left(\frac{dX}{dt}\right)_0$ (s^{-1})[b]	$\left(\frac{-d[LLA]}{dt}\right)$ ($M \cdot s^{-1}$)[c]	k_{obs} (s^{-1})[d]	$k_{obs}[LLA]_0$ ($M \cdot s^{-1}$)[e]	*correction factor*[f]	$(R_{p,corr})_0$ ($M \cdot s^{-1}$)[g]
0.20	2.0	1.0	14.42	35.83	52.28	0.1736	0.0347	0.0802	0.0160	1.195	0.0415
0.22	2.2	1.1	15.10	38.49	55.48	0.1806	0.0397	0.0880	0.0194	1.174	0.0467
0.24	2.4	1.2	15.18	41.08	57.31	0.1787	0.0429	0.0937	0.0225	1.157	0.0496
0.26	2.6	1.3	16.58	45.68	61.23	0.1939	0.0504	0.1061	0.0276	1.143	0.0576
0.28	2.8	1.4	18.17	50.12	65.74	0.2121	0.0594	0.1212	0.0339	1.132	0.0672
0.30	3.0	1.5	18.76	52.57	69.13	0.2182	0.0655	0.1326	0.0398	1.122	0.0734
0.32	3.2	1.6	20.24	56.14	73.97	0.2360	0.0755	0.1513	0.0484	1.113	0.0841
0.34	3.4	1.7	22.27	61.60	77.90	0.2592	0.0881	0.1739	0.0591	1.106	0.0975
0.36	3.6	1.8	24.67	67.02	81.43	0.2878	0.1036	0.1996	0.0719	1.100	0.1139
0.38	3.8	1.9	25.66	71.52	84.93	0.2970	0.1129	0.2274	0.0864	1.094	0.1235
0.40	4.0	2.0	27.24	74.58	88.49	0.3168	0.1267	0.2586	0.1034	1.089	0.1379
0.42	4.2	2.1	30.65	78.34	92.52	0.3623	0.1522	0.3072	0.1290	1.084	0.1650
0.44	4.4	2.2	32.70	83.95	96.26	0.3856	0.1697	0.3727	0.1640	1.080	0.1832
0.46	4.6	2.3	33.31	85.64	97.35	0.3925	0.1805	0.3938	0.1811	1.076	0.1943
0.48	4.8	2.4	34.94	89.88	98.32	0.4109	0.1972	0.4392	0.2108	1.073	0.2116
0.50	5.0	2.5	37.50	93.25	98.85	0.4439	0.2219	0.4846	0.2423	1.070	0.2374
0.52	5.2	2.6	39.17	94.87	99.32	0.4662	0.2424	0.5116	0.2660	1.067	0.2586
0.54	5.4	2.7	39.64	96.29	99.48	0.4712	0.2544	0.5182	0.2798	1.064	0.2708
0.56	5.6	2.8	40.32	95.74	98.40	0.4816	0.2697	0.5222	0.2925	1.062	0.2864
0.58	5.8	2.9	41.27	96.10	99.18	0.4952	0.2872	0.5427	0.3148	1.060	0.3043
0.60	6.0	3.0	42.43	97.77	98.71	0.5098	0.3059	0.5540	0.3324	1.057	0.3234
0.62	6.2	3.1	45.09	97.07	98.84	0.5495	0.3407	0.6001	0.3721	1.056	0.3596
0.64	6.4	3.2	46.45	96.49	99.88	0.5703	0.3650	0.6290	0.4025	1.054	0.3846
0.66	6.6	3.3	46.34	97.90	99.42	0.5668	0.3741	0.6268	0.4137	1.052	0.3935
0.68	6.8	3.4	49.19	98.07	98.14	0.6079	0.4133	0.6486	0.4411	1.050	0.4342
0.70	7.0	3.5	51.80	99.51	99.22	0.6442	0.4510	0.7244	0.5071	1.049	0.4730
0.72	7.2	3.6	51.72	99.27	99.46	0.6434	0.4633	0.7271	0.5235	1.047	0.4852
0.74	7.4	3.7	51.69	98.24	99.46	0.6444	0.4768	0.7276	0.5384	1.046	0.4988
0.76	7.6	3.8	52.58	98.28	99.34	0.6573	0.4995	0.7437	0.5652	1.045	0.5219
0.78	7.8	3.9	53.39	98.96	98.81	0.6681	0.5211	0.7457	0.5817	1.044	0.5438
0.80	8.0	4.0	54.48	99.10	99.14	0.6838	0.5471	0.7778	0.6223	1.042	0.5703

[a] Data from Table S8, Zhang and Junkers, *Chem. Sci.* 2026, **17**, 4706-4714. [b] Derivative of cubic spline interpolator of conversion data at $t = 0$. [c] $\left(\frac{d[LLA]}{dt}\right)_0 = \left(\frac{dX}{dt}\right)_0 \cdot [LLA]_0$. [d] Apparent first-order rate constant obtained by fitting $\hat{X} = 1 - \exp(-k_{obs}t)$ to conversion data. [e] Initial rate of monomer consumption predicted from k_{obs}. [f] Correction factor = $[LLA]_0/([LLA]_0-[LLA]_{eq})$; $[LLA]_{eq}$ = 0.0326 M (Table S18). [g] Corrected $(R_p)_0$ to remove contribution from depolymerization: $(R_{p,corr})_0 = -(d[LLA]/dt)_0 \times [LLA]_0/([LLA]_0-[LLA]_{eq})$.

Table S6. Initial rate of monomer consumption, k_{obs} and depolymerization-corrected rate of monomer consumption for monomer concentration sweep data at 10°C

$[LLA]_0$ (M)[a]	$[MBA]_0$ (mM)[a]	$[TBD]_0$ (mM)[a]	X_1 (%)[a]	X_5 (%)[a]	X_{10} (%)[a]	$\left(\frac{dX}{dt}\right)_0$ (s^{-1})[b]	$\left(\frac{-d[LLA]}{dt}\right)$ ($M \cdot s^{-1}$)[c]	k_{obs} (s^{-1})[d]	$k_{obs}[LLA]_0$ ($M \cdot s^{-1}$)[e]	*correction factor*[f]	$(R_{p,corr})_0$ ($M \cdot s^{-1}$)[g]
0.20	2.0	1.0	24.90	46.88	62.3	0.0126	0.0628	0.1118	0.0224	1.151	0.0722
0.22	2.2	1.1	27.66	50.71	65.94	0.0169	0.0770	0.1252	0.0275	1.135	0.0874
0.24	2.4	1.2	30.29	53.06	68.76	0.0222	0.0925	0.1361	0.0327	1.123	0.1039
0.26	2.6	1.3	33.78	56.23	70.87	0.0293	0.1125	0.1472	0.0383	1.112	0.1251
0.28	2.8	1.4	36.63	60.08	73.44	0.0368	0.1315	0.1617	0.0453	1.103	0.1451
0.30	3.0	1.5	38.86	63.91	77.33	0.0448	0.1494	0.1836	0.0551	1.096	0.1637
0.32	3.2	1.6	42.24	68.88	81.6	0.0555	0.1733	0.2152	0.0689	1.089	0.1888
0.34	3.4	1.7	43.59	73.08	84.65	0.0643	0.1892	0.2450	0.0833	1.083	0.2050
0.36	3.6	1.8	45.49	76.50	87.98	0.0752	0.2089	0.2838	0.1022	1.078	0.2253
0.38	3.8	1.9	46.70	81.57	92.07	0.0855	0.2250	0.3549	0.1349	1.074	0.2416
0.40	4.0	2.0	47.22	86.39	96.4	0.0951	0.2377	0.4658	0.1863	1.070	0.2543
0.42	4.2	2.1	48.79	88.87	99.57	0.1084	0.2581	0.5446	0.2287	1.067	0.2752
0.44	4.4	2.2	50.87	91.40	99.97	0.1242	0.2823	0.6153	0.2707	1.063	0.3001
0.46	4.6	2.3	52.22	94.35	98.47	0.1390	0.3022	0.6765	0.3112	1.060	0.3205
0.48	4.8	2.4	54.44	95.59	98.06	0.1584	0.3301	0.7092	0.3404	1.058	0.3491
0.50	5.0	2.5	56.38	97.29	98.12	0.1784	0.3569	0.7589	0.3795	1.055	0.3766
0.52	5.2	2.6	57.28	99.15	98.99	0.1959	0.3768	0.8310	0.4321	1.053	0.3968
0.54	5.4	2.7	57.90	99.54	99.64	0.2138	0.3960	0.8636	0.4663	1.051	0.4161
0.56	5.6	2.8	58.88	99.20	98.22	0.2345	0.4187	0.8132	0.4554	1.049	0.4393
0.58	5.8	2.9	60.12	98.22	99.10	0.2581	0.4450	0.8934	0.5182	1.047	0.4661
0.60	6.0	3.0	60.41	98.70	99.38	0.2775	0.4625	0.9153	0.5492	1.046	0.4837
0.62	6.2	3.1	61.09	99.50	98.70	0.2997	0.4834	0.8934	0.5539	1.044	0.5047
0.64	6.4	3.2	62.17	99.22	99.09	0.3260	0.5093	0.9449	0.6047	1.043	0.5311
0.66	6.6	3.3	62.96	98.68	99.75	0.3520	0.5334	0.9875	0.6518	1.041	0.5554
0.68	6.8	3.4	63.60	99.04	98.81	0.3777	0.5555	0.9554	0.6497	1.040	0.5777
0.70	7.0	3.5	63.08	99.52	99.23	0.3963	0.5661	0.9755	0.6829	1.039	0.5881
0.72	7.2	3.6	63.47	99.64	98.96	0.4221	0.5862	0.9661	0.6956	1.038	0.6084
0.74	7.4	3.7	64.85	98.91	99.38	0.4575	0.6182	1.0265	0.7596	1.037	0.6409
0.76	7.6	3.8	64.36	98.70	99.80	0.4786	0.6297	1.0252	0.7792	1.036	0.6522
0.78	7.8	3.9	62.76	99.39	99.17	0.4893	0.6273	0.9641	0.7520	1.035	0.6491
0.80	8.0	4.0	64.03	99.29	99.22	0.5266	0.6583	0.9983	0.7986	1.034	0.6806

[a] Data from Table S9, Zhang and Junkers, *Chem. Sci.* 2026, **17**, 4706-4714. [b] Derivative of cubic spline interpolator of conversion data at t = 0. [c] $\left(\frac{d[LLA]}{dt}\right)_0 = \left(\frac{dX}{dt}\right)_0 \cdot [LLA]_0$. [d] Apparent first-order rate constant obtained by fitting $\hat{X} = 1 - \exp(-k_{obs}t)$ to conversion data. [e] Initial rate of monomer consumption predicted from k_{obs}. [f] Correction factor = $[LLA]_0/([LLA]_0-[LLA]_{eq})$; $[LLA]_{eq}$ = 0.0262 M (Table S18). [g] Corrected $(R_p)_0$ to remove contribution from depolymerization: $(R_{p,corr})_0 = -(d[LLA]/dt)_0 \times [LLA]_0/([LLA]_0-[LLA]_{eq})$.

Table S7. Initial rate of monomer consumption, k_{obs} and depolymerization-corrected rate of monomer consumption for monomer concentration sweep data at 0°C

$[LLA]_0$ (M)[a]	$[MBA]_0$ (mM)[a]	$[TBD]_0$ (mM)[a]	X_1 (%)[a]	X_5 (%)[a]	X_{10} (%)[a]	$\left(\frac{dX}{dt}\right)_0$ (s^{-1})[b]	$\left(\frac{-d[LLA]}{dt}\right)$ ($M \cdot s^{-1}$)[c]	k_{obs} (s^{-1})[d]	$k_{obs}[LLA]_0$ ($M \cdot s^{-1}$)[e]	*correction factor*[f]	$(R_{p,corr})_0$ ($M \cdot s^{-1}$)[g]
0.20	2.0	1.0	28.52	59.37	65.67	0.3509	0.0702	0.1324	0.0265	1.115	0.0783
0.22	2.2	1.1	38.68	62.60	67.52	0.4952	0.1089	0.1427	0.0314	1.104	0.1203
0.24	2.4	1.2	44.90	64.39	70.62	0.5841	0.1402	0.1564	0.0375	1.094	0.1534
0.26	2.6	1.3	47.62	66.38	74.78	0.6220	0.1617	0.1765	0.0459	1.087	0.1757
0.28	2.8	1.4	50.52	69.03	80.06	0.6619	0.1853	0.2078	0.0582	1.080	0.2001
0.30	3.0	1.5	53.69	71.71	85.37	0.7056	0.2117	0.2484	0.0745	1.074	0.2274
0.32	3.2	1.6	53.93	74.89	89.85	0.7058	0.2259	0.2965	0.0949	1.069	0.2415
0.34	3.4	1.7	53.60	78.72	96.18	0.6973	0.2371	0.3772	0.1283	1.065	0.2524
0.36	3.6	1.8	53.96	82.41	99.46	0.6983	0.2514	0.4399	0.1584	1.061	0.2667
0.38	3.8	1.9	55.31	86.86	*100*[h]	0.7122	0.2707	*0.5323*[h]	*0.2023*[h]	1.058	0.2862
0.40	4.0	2.0	57.58	92.05	99.83	0.7385	0.2954	0.6933	0.2773	1.055	0.3115
0.42	4.2	2.1	59.34	96.28	99.31	0.7585	0.3186	0.8497	0.3569	1.052	0.3351
0.44	4.4	2.2	59.53	98.35	99.16	0.7585	0.3337	0.8845	0.3892	1.049	0.3502
0.46	4.6	2.3	60.13	98.99	99.88	0.7665	0.3526	0.9193	0.4229	1.047	0.3692
0.48	4.8	2.4	59.64	99.25	99.44	0.7590	0.3643	0.9006	0.4323	1.045	0.3807
0.50	5.0	2.5	61.18	99.35	99.07	0.7812	0.3906	0.9208	0.4604	1.043	0.4075
0.52	5.2	2.6	63.01	99.13	98.90	0.8082	0.4202	0.9503	0.4942	1.041	0.4377
0.54	5.4	2.7	62.70	99.06	98.41	0.8036	0.4340	0.8987	0.4853	1.040	0.4513
0.56	5.6	2.8	63.05	99.45	98.29	0.8082	0.4526	0.8924	0.4998	1.038	0.4700
0.58	5.8	2.9	64.00	99.28	98.15	0.8222	0.4769	0.8923	0.5175	1.037	0.4946
0.60	6.0	3.0	65.30	99.70	99.23	0.8409	0.5045	1.0318	0.6191	1.036	0.5226
0.62	6.2	3.1	64.74	99.91	99.87	0.8326	0.5162	1.0420	0.6460	1.035	0.5340
0.64	6.4	3.2	64.28	99.77	98.97	0.8258	0.5285	0.9858	0.6309	1.033	0.5462
0.66	6.6	3.3	65.42	99.90	98.49	0.8422	0.5559	0.9591	0.6330	1.032	0.5739
0.68	6.8	3.4	65.85	99.84	99.41	0.8487	0.5771	1.0583	0.7196	1.031	0.5953
0.70	7.0	3.5	66.84	99.30	98.36	0.8637	0.6046	0.9679	0.6775	1.030	0.6230
0.72	7.2	3.6	68.09	99.49	97.83	0.8816	0.6347	0.9028	0.6500	1.030	0.6535
0.74	7.4	3.7	66.97	99.71	98.75	0.8651	0.6402	1.0241	0.7578	1.029	0.6586
0.76	7.6	3.8	66.64	99.76	99.10	0.8603	0.6538	1.0555	0.8022	1.028	0.6721
0.78	7.8	3.9	67.49	99.90	98.59	0.8724	0.6805	1.0133	0.7904	1.027	0.6990
0.80	8.0	4.0	64.03	99.69	99.25	0.8224	0.6579	1.0005	0.8004	1.027	0.6754

[a] Data from Table S10, Zhang and Junkers, *Chem. Sci.* 2026, **17**, 4706-4714. [b] Derivative of cubic spline interpolator of conversion data at t = 0. [c] $\left(\frac{d[LLA]}{dt}\right)_0 = \left(\frac{dX}{dt}\right)_0 \cdot [LLA]_0$. [d] Apparent first-order rate constant obtained by fitting $\hat{X} = 1 - \exp(-k_{obs}t)$ to conversion data. [e] Initial rate of monomer consumption predicted from k_{obs}. [f] Correction factor = $[LLA]_0/([LLA]_0-[LLA]_{eq})$; $[LLA]_{eq}$ = 0.0207 M (Table S18). [g] Corrected $(R_p)_0$ to remove contribution from depolymerization: $(R_{p,corr})_0 = -(d[LLA]/dt)_0 \times [LLA]_0/([LLA]_0-[LLA]_{eq})$. [h] X_{10} set to 99.99% to fit k_{obs}.

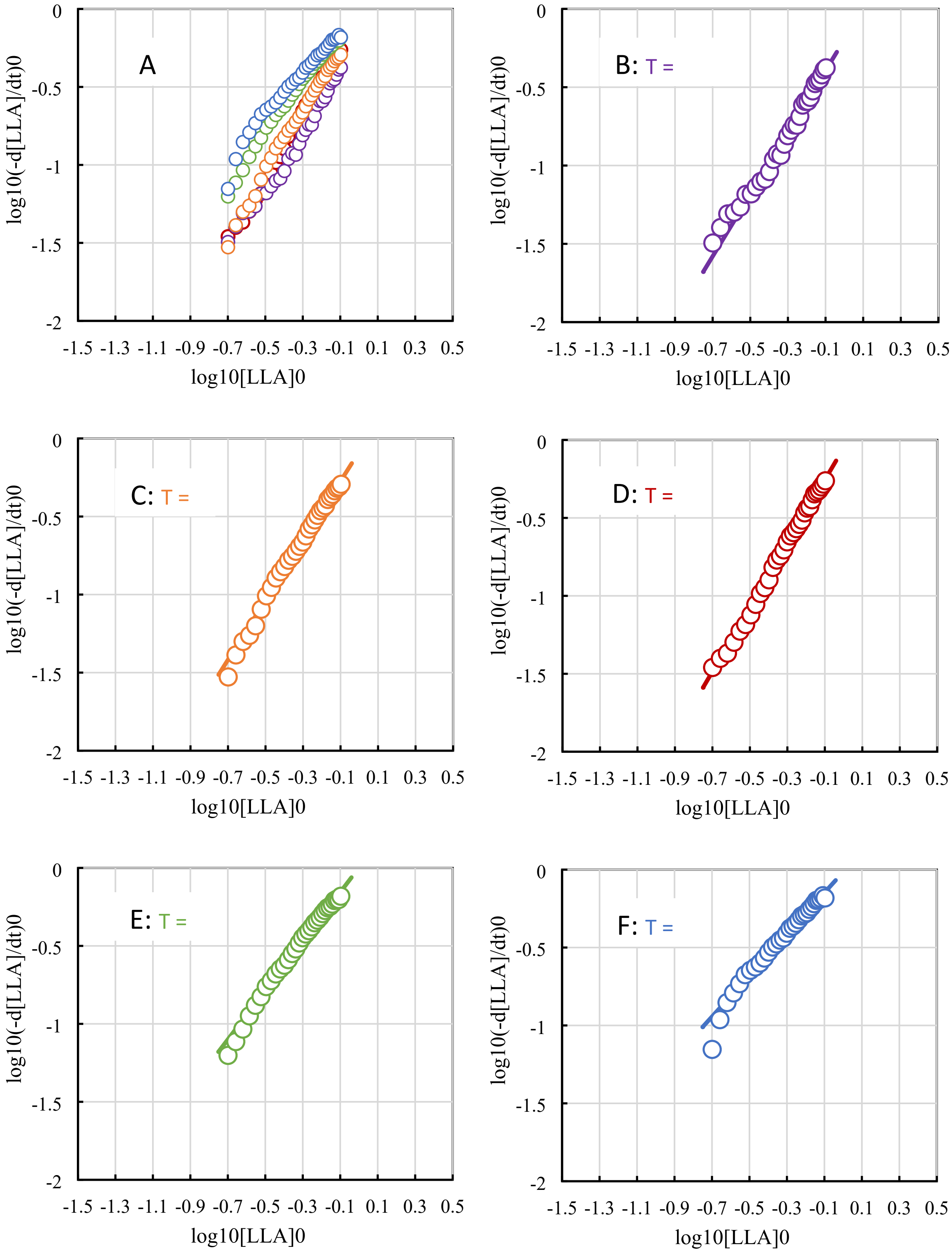

Figure S1. Log-log plots of initial rate of lactide consumption vs initial lactide concentration for all monomer sweep experiments (A) and for individual temperatures showing overall reaction order (B-F).

Table S8. Initial rate of monomer consumption, k_{obs} and depolymerization-corrected rate of monomer consumption for catalyst concentration sweep data at 20°C, $[LLA]_0$ = 0.2 M

$[LLA]_0$ (M)[a]	$[MBA]_0$ (mM)[a]	$[TBD]_0$ (mM)[a]	X_1 (%)[a]	X_5 (%)[a]	X_{10} (%)[a]	$\left(\frac{dX}{dt}\right)_0$ (s^{-1})[b]	$\left(\frac{-d[LLA]}{dt}\right)$ ($M{\cdot}s^{-1}$)[c]	k_{obs} (s^{-1})[d]	$k_{obs}[LLA]_0$ ($M{\cdot}s^{-1}$)[e]	$(R_{p,corr})_0$ ($M{\cdot}s^{-1}$)[f]
0.20	2.0	1.000	10.38	43.21	51.95	0.1049	0.0210	0.0843	0.0169	0.1253
0.20	2.0	0.800	9.60	40.22	47.32	0.0965	0.0193	0.0741	0.0148	0.0922
0.20	2.0	0.667	8.90	39.53	44.23	0.0866	0.0173	0.0685	0.0137	0.0690
0.20	2.0	0.571	8.03	36.96	42.90	0.0770	0.0154	0.0649	0.0130	0.0526
0.20	2.0	0.500	7.37	35.85	40.99	0.0685	0.0137	0.0613	0.0123	0.0409
0.20	2.0	0.444	6.99	32.21	37.06	0.0669	0.0134	0.0537	0.0107	0.0355
0.20	2.0	0.400	6.92	30.60	33.50	0.0673	0.0135	0.0481	0.0096	0.0322
0.20	2.0	0.364	6.69	27.96	31.31	0.0670	0.0134	0.0440	0.0088	0.0291
0.20	2.0	0.333	6.12	25.13	30.06	0.0622	0.0124	0.0410	0.0082	0.0248
0.20	2.0	0.308	6.03	22.80	27.04	0.0633	0.0127	0.0363	0.0073	0.0233
0.20	2.0	0.286	6.18	21.73	25.18	0.0665	0.0133	0.0337	0.0067	0.0227
0.20	2.0	0.267	5.49	21.17	22.38	0.0567	0.0113	0.0302	0.0060	0.0181
0.20	2.0	0.250	5.74	19.58	21.18	0.0622	0.0124	0.0282	0.0056	0.0186
0.20	2.0	0.235	6.01	17.71	20.60	0.0684	0.0137	0.0268	0.0054	0.0192
0.20	2.0	0.222	6.05	16.51	19.90	0.0705	0.0141	0.0256	0.0051	0.0187
0.20	2.0	0.211	5.57	15.69	19.49	0.0645	0.0129	0.0247	0.0049	0.0162
0.20	2.0	0.200	5.46	16.19	18.19	0.0619	0.0124	0.0236	0.0047	0.0148
0.20	2.0	0.190	4.73	15.28	17.05	0.0523	0.0105	0.0220	0.0044	0.0119
0.20	2.0	0.182	4.51	13.61	16.08	0.0511	0.0102	0.0203	0.0041	0.0111
0.20	2.0	0.174	5.42	12.12	17.37	0.0666	0.0133	0.0209	0.0042	0.0138
0.20	2.0	0.167	4.68	11.58	16.53	0.0563	0.0113	0.0198	0.0040	0.0112

[a] Data from Table S11, Zhang and Junkers, *Chem. Sci.* 2026, **17**, 4706-4714. [b] Derivative of cubic spline interpolator of conversion data at t = 0. [c] $\left(\frac{d[LLA]}{dt}\right)_0 = \left(\frac{dX}{dt}\right)_0 \cdot [LLA]_0$. [d] Apparent first-order rate constant obtained by fitting $\hat{X} = 1 - \exp(-k_{obs}t)$ to conversion data. [e] Initial rate of monomer consumption predicted from k_{obs}. [f] Corrected $(R_p)_0$ to remove contribution from depolymerization: $(R_{p,corr})_0$ = $-(d[LLA]/dt)_0 \times [LLA]_0/([LLA]_0-[LLA]_{eq}) = -(d[LLA]/dt)_0 \times 1.195$; $[LLA]_{eq}$ = 0.0326 M (Table S18).

Table S9. Initial rate of monomer consumption, k_{obs} and depolymerization-corrected rate of monomer consumption for catalyst concentration sweep data at 20°C, $[LLA]_0$ = 0.35 M

$[LLA]_0$ (M)[a]	$[MBA]_0$ (mM)[a]	$[TBD]_0$ (mM)[a]	X_1 (%)[a]	X_5 (%)[a]	X_{10} (%)[a]	$\left(\frac{dX}{dt}\right)_0$ (s^{-1})[b]	$\left(\frac{-d[LLA]}{dt}\right)_0$ ($M \cdot s^{-1}$)[c]	k_{obs} (s^{-1})[d]	$k_{obs}[LLA]_0$ ($M \cdot s^{-1}$)[e]	$(R_{p,corr})_0$ ($M \cdot s^{-1}$)[f]
0.35	3.5	1.750	36.95	68.21	80.18	0.4651	0.1628	0.2041	0.0714	0.1795
0.35	3.5	1.400	33.89	64.40	77.35	0.4249	0.1487	0.1828	0.0640	0.1640
0.35	3.5	1.167	29.87	60.02	71.45	0.3709	0.1298	0.1526	0.0534	0.1431
0.35	3.5	1.000	27.74	58.60	66.13	0.3406	0.1192	0.1331	0.0466	0.1315
0.35	3.5	0.875	25.12	55.10	60.29	0.3058	0.1070	0.1135	0.0397	0.1180
0.35	3.5	0.778	22.85	51.39	57.92	0.2771	0.0970	0.1049	0.0367	0.1070
0.35	3.5	0.700	20.28	48.27	54.29	0.2431	0.0851	0.0943	0.0330	0.0938
0.35	3.5	0.636	17.43	45.28	49.32	0.2044	0.0716	0.0821	0.0287	0.0789
0.35	3.5	0.583	15.67	42.39	45.07	0.1817	0.0636	0.0726	0.0254	0.0701
0.35	3.5	0.538	13.41	38.82	42.57	0.1530	0.0535	0.0663	0.0232	0.0590
0.35	3.5	0.500	12.99	35.00	38.42	0.1510	0.0529	0.0580	0.0203	0.0583
0.35	3.5	0.467	12.03	31.92	35.81	0.1406	0.0492	0.0526	0.0184	0.0543
0.35	3.5	0.438	11.35	28.04	33.77	0.1354	0.0474	0.0478	0.0167	0.0522
0.35	3.5	0.412	11.31	24.36	31.29	0.1391	0.0487	0.0428	0.0150	0.0537
0.35	3.5	0.389	11.19	22.16	29.24	0.1399	0.0490	0.0392	0.0137	0.0540
0.35	3.5	0.368	9.60	23.03	29.03	0.1155	0.0404	0.0392	0.0137	0.0446
0.35	3.5	0.350	8.51	22.82	27.97	0.0997	0.0349	0.0377	0.0132	0.0385
0.35	3.5	0.333	10.18	21.66	27.11	0.1254	0.0439	0.0363	0.0127	0.0484
0.35	3.5	0.318	8.94	21.32	26.19	0.1075	0.0376	0.0350	0.0122	0.0415
0.35	3.5	0.304	9.60	19.98	27.34	0.1192	0.0417	0.0357	0.0125	0.0460
0.35	3.5	0.292	9.34	18.86	25.93	0.1166	0.0408	0.0335	0.0117	0.0450

[a] Data from Table S12, Zhang and Junkers, *Chem. Sci.* 2026, **17**, 4706-4714. [b] Derivative of cubic spline interpolator of conversion data at t = 0. [c] $\left(\frac{d[LLA]}{dt}\right)_0 = \left(\frac{dX}{dt}\right)_0 \cdot [LLA]_0$. [d] Apparent first-order rate constant obtained by fitting $\hat{X} = 1 - \exp(-k_{obs}t)$ to conversion data. [e] Initial rate of monomer consumption predicted from k_{obs}. [f] Corrected $(R_p)_0$ to remove contribution from depolymerization: $(R_{p,corr})_0$ = $-(d[LLA]/dt)_0 \times [LLA]_0/([LLA]_0-[LLA]_{eq}) = -(d[LLA]/dt)_0 \times 1.103$; $[LLA]_{eq}$ = 0.0326 M (Table S18).

Table S10. Initial rate of monomer consumption, k_{obs} and depolymerization-corrected rate of monomer consumption for catalyst concentration sweep data at 20°C, $[LLA]_0$ = 0.5 M

$[LLA]_0$ (M)[a]	$[MBA]_0$ (mM)[a]	$[TBD]_0$ (mM)[a]	X_1 (%)[a]	X_5 (%)[a]	X_{10} (%)[a]	$\left(\frac{dX}{dt}\right)_0$ (s^{-1})[b]	$\left(\frac{-d[LLA]}{dt}\right)_0$ ($M \cdot s^{-1}$)[c]	k_{obs} (s^{-1})[d]	$k_{obs}[LLA]_0$ ($M \cdot s^{-1}$)[e]	$(R_{p,corr})_0$ ($M \cdot s^{-1}$)[f]
0.50	5.0	2.500	52.09	99.95	100[g]	0.6480	0.3240	*0.7359*[g]	*0.3679*[g]	0.3466
0.50	5.0	2.000	50.37	99.73	100[g]	0.6233	0.3116	*0.7009*[g]	*0.3505*[g]	0.3334
0.50	5.0	1.667	47.57	99.23	100[g]	0.5831	0.2915	*0.6474*[g]	*0.3237*[g]	0.3119
0.50	5.0	1.429	41.00	93.61	100[g]	0.4947	0.2473	*0.5327*[g]	*0.2664*[g]	0.2646
0.50	5.0	1.250	38.06	90.80	100[g]	0.4555	0.2277	*0.4784*[g]	*0.2392*[g]	0.2436
0.50	5.0	1.111	35.75	85.26	98.99	0.4289	0.2144	0.4091	0.2046	0.2294
0.50	5.0	1.000	33.92	76.86	93.21	0.4121	0.2061	0.3116	0.1558	0.2204
0.50	5.0	0.909	32.79	71.06	87.40	0.4021	0.2011	0.2486	0.1243	0.2151
0.50	5.0	0.833	31.39	68.32	83.81	0.3846	0.1923	0.2199	0.1099	0.2057
0.50	5.0	0.769	27.46	61.69	79.62	0.3352	0.1676	0.1837	0.0918	0.1793
0.50	5.0	0.714	26.83	57.20	73.40	0.3306	0.1653	0.1543	0.0772	0.1769
0.50	5.0	0.667	26.26	54.56	67.26	0.3246	0.1623	0.1325	0.0663	0.1736
0.50	5.0	0.625	26.81	52.57	62.34	0.3342	0.1671	0.1175	0.0588	0.1788
0.50	5.0	0.588	27.06	50.37	59.61	0.3402	0.1701	0.1091	0.0545	0.1820
0.50	5.0	0.556	25.37	46.39	57.17	0.3203	0.1602	0.1002	0.0501	0.1713
0.50	5.0	0.526	25.81	40.66	50.35	0.3329	0.1665	0.0826	0.0413	0.1781
0.50	5.0	0.500	24.75	38.23	46.18	0.3198	0.1599	0.0736	0.0368	0.1711
0.50	5.0	0.476	24.21	34.65	44.91	0.3164	0.1582	0.0692	0.0346	0.1692
0.50	5.0	0.455	23.53	32.53	43.97	0.3091	0.1546	0.0662	0.0331	0.1653
0.50	5.0	0.435	22.79	29.49	39.94	0.3015	0.1508	0.0583	0.0291	0.1613
0.50	5.0	0.417	21.70	29.24	37.77	0.2855	0.1428	0.0550	0.0275	0.1527

[a] Data from Table S13, Zhang and Junkers, *Chem. Sci.* 2026, **17**, 4706-4714. [b] Derivative of cubic spline interpolator of conversion data at t = 0. [c] $\left(\frac{d[LLA]}{dt}\right)_0 = \left(\frac{dX}{dt}\right)_0 \cdot [LLA]_0$. [d] Apparent first-order rate constant obtained by fitting $\hat{X} = 1 - \exp\left(-k_{obs}t\right)$ to conversion data. [e] Initial rate of monomer consumption predicted from k_{obs}. [f] Corrected $(R_p)_0$ to remove contribution from depolymerization: $(R_{p,corr})_0$ = $-(d[LLA]/dt)_0 \times [LLA]_0/([LLA]_0-[LLA]_{eq}) = -(d[LLA]/dt)_0 \times 1.070$; $[LLA]_{eq}$ = 0.0326 M (Table S18). [g] X_{10} set to 99.99% to fit k_{obs}.

Table S11. Initial rate of monomer consumption, k_{obs} and depolymerization-corrected rate of monomer consumption for catalyst concentration sweep data at 20°C, $[LLA]_0$ = 0.7 M

$[LLA]_0$ (M)[a]	$[MBA]_0$ (mM)[a]	$[TBD]_0$ (mM)[a]	X_1 (%)[a]	X_5 (%)[a]	X_{10} (%)[a]	$\left(\frac{dX}{dt}\right)_0$ (s^{-1})[b]	$\left(\frac{-d[LLA]}{dt}\right)_0$ ($M \cdot s^{-1}$)[c]	k_{obs} (s^{-1})[d]	$k_{obs}[LLA]_0$ ($M \cdot s^{-1}$)[e]	$(R_{p,corr})_0$ ($M \cdot s^{-1}$)[f]
0.70	7.0	3.500	62.23	97.11	100[g]	0.7997	0.5598	*0.9398*[g]	*0.6579*[g]	0.5871
0.70	7.0	2.800	60.83	96.66	100[g]	0.7798	0.5459	*0.8977*[g]	*0.6284*[g]	0.5726
0.70	7.0	2.333	57.02	93.00	96.15	0.7283	0.5098	0.5915	0.4141	0.5347
0.70	7.0	2.000	53.66	89.34	93.11	0.6835	0.4785	0.4308	0.3015	0.5018
0.70	7.0	1.750	50.61	86.68	90.5	0.6420	0.4494	0.3543	0.2480	0.4714
0.70	7.0	1.556	48.80	83.98	88.38	0.6188	0.4331	0.3111	0.2177	0.4543
0.70	7.0	1.400	47.38	81.77	86.02	0.6005	0.4203	0.2760	0.1932	0.4409
0.70	7.0	1.273	45.67	78.32	84.85	0.5799	0.4059	0.2583	0.1808	0.4257
0.70	7.0	1.167	45.03	76.79	82.21	0.5720	0.4004	0.2328	0.1630	0.4200
0.70	7.0	1.077	44.57	74.16	79.50	0.5682	0.3978	0.2102	0.1472	0.4172
0.70	7.0	1.000	43.75	71.42	78.20	0.5596	0.3917	0.1993	0.1395	0.4109
0.70	7.0	0.933	42.60	69.05	76.10	0.5456	0.3819	0.1852	0.1296	0.4005
0.70	7.0	0.875	41.11	66.31	72.68	0.5267	0.3687	0.1662	0.1163	0.3867
0.70	7.0	0.824	39.23	62.66	68.82	0.5034	0.3524	0.1476	0.1033	0.3696
0.70	7.0	0.778	38.50	59.96	64.83	0.4955	0.3468	0.1319	0.0923	0.3638
0.70	7.0	0.737	37.34	57.10	61.93	0.4817	0.3372	0.1212	0.0848	0.3537
0.70	7.0	0.700	35.23	53.57	59.09	0.4551	0.3185	0.1111	0.0778	0.3341
0.70	7.0	0.667	34.01	49.65	55.89	0.4418	0.3092	0.1008	0.0705	0.3244
0.70	7.0	0.636	33.75	46.93	54.00	0.4412	0.3088	0.0948	0.0664	0.3239
0.70	7.0	0.609	32.05	43.63	52.68	0.4205	0.2943	0.0898	0.0628	0.3087
0.70	7.0	0.583	30.81	41.64	51.91	0.4049	0.2834	0.0868	0.0607	0.2973

[a] Data from Table S13, Zhang and Junkers, *Chem. Sci.* 2026, **17**, 4706-4714. [b] Derivative of cubic spline interpolator of conversion data at t = 0. [c] $\left(\frac{d[LLA]}{dt}\right)_0 = \left(\frac{dX}{dt}\right)_0 \cdot [LLA]_0$. [d] Apparent first-order rate constant obtained by fitting $\hat{X} = 1 - \exp\left(-k_{obs}t\right)$ to conversion data. [e] Initial rate of monomer consumption predicted from k_{obs}. [f] Corrected $(R_p)_0$ to remove contribution from depolymerization: $(R_{p,corr})_0$ = $-(d[LLA]/dt)_0 \times [LLA]_0/([LLA]_0-[LLA]_{eq}) = -(d[LLA]/dt)_0 \times 1.050$; $[LLA]_{eq}$ = 0.0326 M (Table S18). [g] X_{10} set to 99.99% to fit k_{obs}.

Table S12. Initial rate of monomer consumption, k_{obs} and depolymerization-corrected rate of monomer consumption for degree of polymerization sweep data at 20°C, $[LLA]_0$ = 0.2 M

$[LLA]_0$ (M)[a]	$[MBA]_0$ (mM)[a]	$[TBD]_0$ (mM)[a]	X_1 (%)[a]	X_5 (%)[a]	X_{10} (%)[a]	$\left(\frac{dX}{dt}\right)_0$ (s^{-1})[b]	$\left(\frac{-d[LLA]}{dt}\right)_0$ ($M \cdot s^{-1}$)[c]	k_{obs} (s^{-1})[d]	$k_{obs}[LLA]_0$ ($M \cdot s^{-1}$)[e]	$(R_{p,corr})_0$ ($M \cdot s^{-1}$)[f]
0.20	4.000	0.667	11.54	48.51	63.88	0.1172	0.0234	0.1126	0.0225	0.0280
0.20	3.636	0.667	10.97	47.15	60.36	0.1100	0.0220	0.1037	0.0207	0.0263
0.20	3.333	0.667	11.29	46.49	56.70	0.1148	0.0230	0.0959	0.0192	0.0274
0.20	3.077	0.667	8.60	43.20	54.62	0.0795	0.0159	0.0886	0.0177	0.0190
0.20	2.857	0.667	7.56	40.33	53.99	0.0679	0.0136	0.0850	0.0170	0.0162
0.20	2.667	0.667	8.57	38.74	51.26	0.0842	0.0168	0.0795	0.0159	0.0201
0.20	2.500	0.667	7.63	38.20	51.63	0.0713	0.0143	0.0795	0.0159	0.0170
0.20	2.353	0.667	9.91	36.28	49.28	0.1066	0.0213	0.0748	0.0150	0.0255
0.20	2.222	0.667	7.79	35.41	47.56	0.0765	0.0153	0.0711	0.0142	0.0183
0.20	2.105	0.667	7.72	32.87	50.19	0.0793	0.0159	0.0730	0.0146	0.0190
0.20	2.000	0.667	6.55	30.64	48.97	0.0650	0.0130	0.0691	0.0138	0.0155
0.20	1.905	0.667	8.59	30.16	46.70	0.0949	0.0190	0.0661	0.0132	0.0227
0.20	1.818	0.667	6.60	28.97	48.37	0.0678	0.0136	0.0669	0.0134	0.0162
0.20	1.739	0.667	8.55	27.99	46.22	0.0971	0.0194	0.0637	0.0127	0.0232
0.20	1.667	0.667	6.64	26.28	47.17	0.0717	0.0143	0.0630	0.0126	0.0171
0.20	1.600	0.667	7.22	25.96	44.39	0.0800	0.0160	0.0594	0.0119	0.0191
0.20	1.538	0.667	8.73	25.46	45.45	0.1029	0.0206	0.0606	0.0121	0.0246
0.20	1.481	0.667	7.50	24.05	43.61	0.0864	0.0173	0.0570	0.0114	0.0207
0.20	1.429	0.667	8.17	25.17	40.63	0.0941	0.0188	0.0543	0.0109	0.0225
0.20	1.379	0.667	10.20	25.44	38.27	0.1229	0.0246	0.0519	0.0104	0.0294
0.20	1.333	0.667	9.94	23.12	36.64	0.1218	0.0244	0.0483	0.0097	0.0291

[a] Data from Table S17, Zhang and Junkers, *Chem. Sci.* 2026, **17**, 4706-4714. [b] Derivative of cubic spline interpolator of conversion data at t = 0. [c] $\left(\frac{d[LLA]}{dt}\right)_0 = \left(\frac{dX}{dt}\right)_0 \cdot [LLA]_0$. [d] Apparent first-order rate constant obtained by fitting $\hat{X} = 1 - \exp(-k_{obs}t)$ to conversion data. [e] Initial rate of monomer consumption predicted from k_{obs}. [f] Corrected $(R_p)_0$ to remove contribution from depolymerization: $(R_{p,corr})_0$ = $-(d[LLA]/dt)_0 \times [LLA]_0/([LLA]_0-[LLA]_{eq}) = -(d[LLA]/dt)_0 \times 1.195$; $[LLA]_{eq}$ = 0.0326 M (Table S18).

Table S13. Initial rate of monomer consumption, k_{obs} and depolymerization-corrected rate of monomer consumption for degree of polymerization sweep data at 20°C, $[LLA]_0$ = 0.35 M

$[LLA]_0$ (M)[a]	$[MBA]_0$ (mM)[a]	$[TBD]_0$ (mM)[a]	X_1 (%)[a]	X_5 (%)[a]	X_{10} (%)[a]	$\left(\frac{dX}{dt}\right)_0$ (s^{-1})[b]	$\left(\frac{-d[LLA]}{dt}\right)_0$ ($M \cdot s^{-1}$)[c]	k_{obs} (s^{-1})[d]	$k_{obs}[LLA]_0$ ($M \cdot s^{-1}$)[e]	$(R_{p,corr})_0$ ($M \cdot s^{-1}$)[f]
0.35	7.000	1.167	23.42	57.56	73.85	0.2805	0.0982	0.1547	0.0542	0.1083
0.35	6.364	1.167	23.24	57.10	72.40	0.2782	0.0974	0.1495	0.0523	0.1074
0.35	5.833	1.167	22.98	54.99	71.65	0.2771	0.0970	0.1443	0.0505	0.1069
0.35	5.385	1.167	22.72	53.59	71.52	0.2751	0.0963	0.1419	0.0497	0.1062
0.35	5.000	1.167	22.32	54.07	70.60	0.2684	0.0939	0.1399	0.0490	0.1036
0.35	4.667	1.167	21.87	52.55	69.28	0.2636	0.0923	0.1342	0.0470	0.1017
0.35	4.375	1.167	21.10	51.85	68.74	0.2532	0.0886	0.1316	0.0461	0.0977
0.35	4.118	1.167	20.44	49.33	67.82	0.2467	0.0863	0.1259	0.0441	0.0952
0.35	3.889	1.167	19.88	48.98	67.14	0.2389	0.0836	0.1237	0.0433	0.0922
0.35	3.684	1.167	19.37	47.86	66.32	0.2327	0.0815	0.1203	0.0421	0.0898
0.35	3.500	1.167	18.69	47.18	65.50	0.2235	0.0782	0.1174	0.0411	0.0863
0.35	3.333	1.167	18.19	46.36	65.06	0.2172	0.0760	0.1153	0.0404	0.0838
0.35	3.182	1.167	17.09	45.74	64.28	0.2019	0.0707	0.1126	0.0394	0.0779
0.35	3.043	1.167	16.61	44.73	63.69	0.1961	0.0686	0.1100	0.0385	0.0757
0.35	2.917	1.167	16.21	43.60	63.04	0.1916	0.0671	0.1073	0.0376	0.0739
0.35	2.800	1.167	15.69	42.81	62.33	0.1849	0.0647	0.1049	0.0367	0.0714
0.35	2.692	1.167	15.08	42.16	60.35	0.1765	0.0618	0.1003	0.0351	0.0681
0.35	2.593	1.167	14.60	41.85	59.03	0.1696	0.0594	0.0974	0.0341	0.0655
0.35	2.500	1.167	14.27	40.96	56.63	0.1655	0.0579	0.0921	0.0322	0.0639
0.35	2.414	1.167	14.12	39.02	55.62	0.1657	0.0580	0.0885	0.0310	0.0639
0.35	2.333	1.167	13.89	37.86	54.13	0.1635	0.0572	0.0849	0.0297	0.0631

[a] Data from Table S18, Zhang and Junkers, *Chem. Sci.* 2026, **17**, 4706-4714. [b] Derivative of cubic spline interpolator of conversion data at *t* = 0. [c] $\left(\frac{d[LLA]}{dt}\right)_0 = \left(\frac{dX}{dt}\right)_0 \cdot [LLA]_0$. [d] Apparent first-order rate constant obtained by fitting $\hat{X} = 1 - \exp(-k_{obs}t)$ to conversion data. [e] Initial rate of monomer consumption predicted from k_{obs}. [f] Corrected $(R_p)_0$ to remove contribution from depolymerization: $(R_{p,corr})_0$ = $-(d[LLA]/dt)_0 \times [LLA]_0/([LLA]_0-[LLA]_{eq}) = -(d[LLA]/dt)_0 \times 1.103$; $[LLA]_{eq}$ = 0.0326 M (Table S18).

Table S14. Initial rate of monomer consumption, k_{obs} and depolymerization-corrected rate of monomer consumption for degree of polymerization sweep data at 20°C, $[LLA]_0$ = 0.5 M

$[LLA]_0$ (M)[a]	$[MBA]_0$ (mM)[a]	$[TBD]_0$ (mM)[a]	X_1 (%)[a]	X_5 (%)[a]	X_{10} (%)[a]	$\left(\frac{dX}{dt}\right)_0$ (s^{-1})[b]	$\left(\frac{-d[LLA]}{dt}\right)_0$ ($M \cdot s^{-1}$)[c]	k_{obs} (s^{-1})[d]	$k_{obs}[LLA]_0$ ($M \cdot s^{-1}$)[e]	$(R_{p,corr})_0$ ($M \cdot s^{-1}$)[f]
0.50	10.00	1.667	31.35	65.82	93.03	0.3891	0.1946	0.2408	0.1204	0.2081
0.50	9.091	1.667	30.97	63.13	92.84	0.3871	0.1935	0.2256	0.1128	0.2070
0.50	8.333	1.667	30.96	62.70	91.75	0.3873	0.1936	0.2227	0.1114	0.2072
0.50	7.692	1.667	29.99	59.78	90.06	0.3767	0.1883	0.2064	0.1032	0.2015
0.50	7.143	1.667	29.06	57.40	87.71	0.3658	0.1829	0.1929	0.0965	0.1956
0.50	6.667	1.667	28.32	55.03	86.46	0.3578	0.1789	0.1818	0.0909	0.1914
0.50	6.250	1.667	28.38	56.19	88.85	0.3577	0.1788	0.1886	0.0943	0.1913
0.50	5.882	1.667	28.75	53.64	86.65	0.3660	0.1830	0.1771	0.0885	0.1958
0.50	5.556	1.667	27.83	54.63	84.59	0.3508	0.1754	0.1775	0.0887	0.1877
0.50	5.263	1.667	27.82	54.02	81.30	0.3508	0.1754	0.1696	0.0848	0.1877
0.50	5.000	1.667	26.90	53.48	82.17	0.3383	0.1692	0.1692	0.0846	0.1810
0.50	4.762	1.667	27.29	54.27	78.49	0.3422	0.1711	0.1635	0.0817	0.1830
0.50	4.545	1.667	25.29	53.06	79.19	0.3148	0.1574	0.1612	0.0806	0.1684
0.50	4.348	1.667	24.25	50.51	78.53	0.3028	0.1514	0.1530	0.0765	0.1620
0.50	4.167	1.667	25.49	49.71	77.60	0.3218	0.1609	0.1499	0.0750	0.1721
0.50	4.000	1.667	24.35	50.85	76.99	0.3035	0.1518	0.1508	0.0754	0.1624
0.50	3.846	1.667	24.15	51.00	75.59	0.3002	0.1501	0.1480	0.0740	0.1605
0.50	3.704	1.667	24.15	51.17	74.22	0.2997	0.1498	0.1452	0.0726	0.1603
0.50	3.571	1.667	22.73	51.77	73.29	0.2780	0.1390	0.1435	0.0717	0.1487
0.50	3.448	1.667	21.14	52.00	72.75	0.2544	0.1272	0.1419	0.0709	0.1361
0.50	3.333	1.667	18.76	51.67	69.51	0.2195	0.1097	0.1323	0.0662	0.1174

[a] Data from Table S19, Zhang and Junkers, *Chem. Sci.* 2026, **17**, 4706-4714. [b] Derivative of cubic spline interpolator of conversion data at t = 0. [c] $\left(\frac{d[LLA]}{dt}\right)_0 = \left(\frac{dX}{dt}\right)_0 \cdot [LLA]_0$. [d] Apparent first-order rate constant obtained by fitting $\hat{X} = 1 - \exp(-k_{obs}t)$ to conversion data. [e] Initial rate of monomer consumption predicted from k_{obs}. [f] Corrected $(R_p)_0$ to remove contribution from depolymerization: $(R_{p,corr})_0$ = $-(d[LLA]/dt)_0 \times [LLA]_0/([LLA]_0-[LLA]_{eq}) = -(d[LLA]/dt)_0 \times 1.070$; $[LLA]_{eq}$ = 0.0326 M (Table S18).

Table S15. Initial rate of monomer consumption, k_{obs} and depolymerization-corrected rate of monomer consumption for degree of polymerization sweep data at 20°C, $[LLA]_0$ = 0.7 M

$[LLA]_0$ (M)[a]	$[MBA]_0$ (mM)[a]	$[TBD]_0$ (mM)[a]	X_1 (%)[a]	X_5 (%)[a]	X_{10} (%)[a]	$\left(\frac{dX}{dt}\right)_0$ (s^{-1})[b]	$\left(\frac{-d[LLA]}{dt}\right)_0$ ($M{\cdot}s^{-1}$)[c]	k_{obs} (s^{-1})[d]	$k_{obs}[LLA]_0$ ($M{\cdot}s^{-1}$)[e]	$(R_{p,corr})_0$ ($M{\cdot}s^{-1}$)[f]
0.70	14.00	2.333	47.19	89.34	98.31	0.5902	0.4132	0.5357	0.3750	0.4333
0.70	12.73	2.333	46.85	87.8	98.04	0.5872	0.4111	0.5054	0.3537	0.4311
0.70	11.67	2.333	44.86	85.63	97.85	0.5610	0.3927	0.4605	0.3224	0.4119
0.70	10.77	2.333	42.57	84.34	97.37	0.5292	0.3705	0.4302	0.3012	0.3886
0.70	10.00	2.333	43.13	84.21	97.06	0.5375	0.3763	0.4289	0.3002	0.3946
0.70	9.333	2.333	42.75	83.49	96.60	0.5328	0.3730	0.4155	0.2909	0.3912
0.70	8.750	2.333	41.83	83.13	96.39	0.5198	0.3639	0.4066	0.2846	0.3817
0.70	8.235	2.333	39.76	82.14	96.14	0.4909	0.3436	0.3873	0.2711	0.3604
0.70	7.778	2.333	39.82	81.75	95.85	0.4922	0.3446	0.3818	0.2673	0.3614
0.70	7.368	2.333	38.68	81.56	95.53	0.4758	0.3331	0.3741	0.2619	0.3493
0.70	7.000	2.333	37.00	80.81	95.27	0.4522	0.3166	0.3605	0.2523	0.3320
0.70	6.667	2.333	37.43	79.47	95.58	0.4603	0.3222	0.3524	0.2467	0.3380
0.70	6.364	2.333	35.07	78.97	95.31	0.4265	0.2986	0.3401	0.2380	0.3132
0.70	6.087	2.333	34.91	78.22	95.14	0.4251	0.2976	0.3331	0.2332	0.3121
0.70	5.833	2.333	35.21	76.68	94.69	0.4315	0.3020	0.3206	0.2244	0.3168
0.70	5.600	2.333	35.08	75.12	94.37	0.4315	0.3021	0.3079	0.2155	0.3168
0.70	5.385	2.333	33.75	74.79	94.50	0.4126	0.2888	0.3031	0.2122	0.3029
0.70	5.185	2.333	31.83	74.43	94.54	0.3851	0.2696	0.2964	0.2075	0.2827
0.70	5.000	2.333	31.52	75.38	94.46	0.3793	0.2655	0.3016	0.2112	0.2785
0.70	4.828	2.333	30.18	73.03	94.28	0.3628	0.2540	0.2831	0.1981	0.2664
0.70	4.667	2.333	29.57	74.68	94.41	0.3518	0.2462	0.2921	0.2045	0.2583

[a] Data from Table S20, Zhang and Junkers, *Chem. Sci.* 2026, **17**, 4706-4714. [b] Derivative of cubic spline interpolator of conversion data at t = 0. [c] $\left(\frac{d[LLA]}{dt}\right)_0 = \left(\frac{dX}{dt}\right)_0 \cdot [LLA]_0$. [d] Apparent first-order rate constant obtained by fitting $\hat{X} = 1 - \exp(-k_{obs}t)$ to conversion data. [e] Initial rate of monomer consumption predicted from k_{obs}. [f] Corrected $(R_p)_0$ to remove contribution from depolymerization: $(R_{p,corr})_0$ = $-(d[LLA]/dt)_0 \times [LLA]_0/([LLA]_0-[LLA]_{eq}) = -(d[LLA]/dt)_0 \times 1.049$; $[LLA]_{eq}$ = 0.0326 M (Table S18).

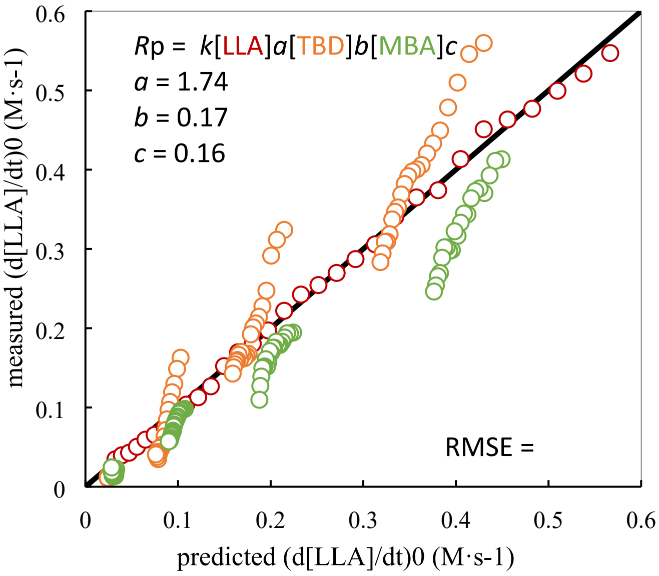

Figure S2. Comparison of predicted and measured initial rates for all experiments fit simultaneously. Red: monomer concentration sweep (Tables S3-S7); orange: catalyst-to-monomer sweep (Tables S8-S10); green: DP sweep (Tables S11-S15).

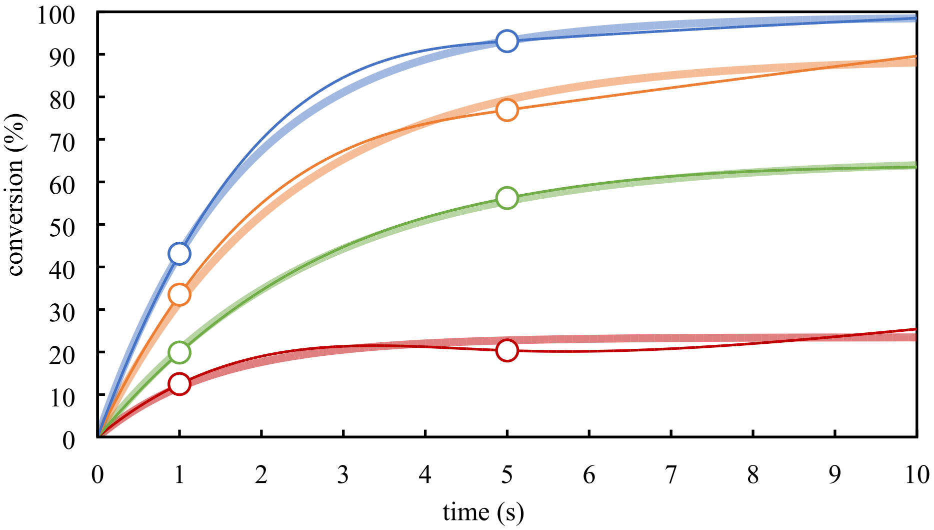

Figure S3. Conversion data from monomer concentration sweep experiments at 35°C, $[LLA]_0$ = 0.2 M (red), 0.4 M (green), 0.6 M (orange) and 0.8 M (blue), [LLA]:[MBA]:[TBD] = 100:1:0.5. Broad lines show best fit to a first-order model incorporating a reversible propagation step with equilibrium monomer concentration $[LLA]_{eq}$ (conversion = $(1-[LLA]_{eq}/[LLA]_0)(1-\exp(-k_{obs}\cdot t))$). Narrow lines show cubic spline fit.

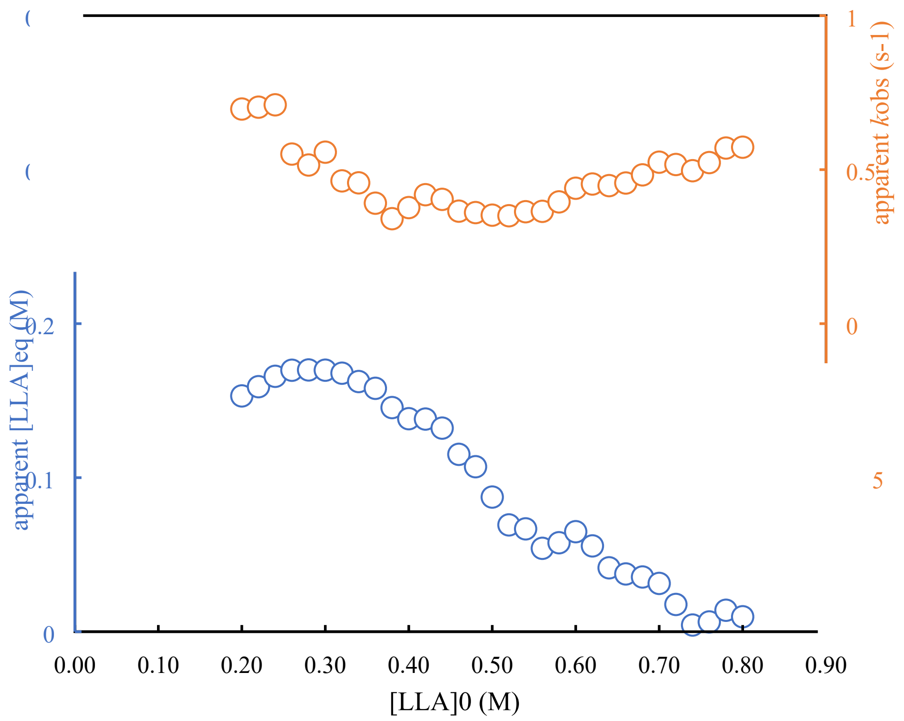

Figure S4. Fitted values of k_{obs} (orange, scale at right) and $[LLA]_{eq}$ (blue, scale at left) as a function of initial LLA concentration $[LLA]_0$. Values obtained by fitting conversion data from monomer sweep experiments at 35°C (Table S3) to a first order kinetic model with a reversible depropagation step: conversion = $(1-[LLA]_{eq}/[LLA]_0)(1- \exp(-k_{obs}\cdot t))$.

Table S16. Apparent first-order rate constant and equilibrium L-lactide concentration for monomer concentration sweep data at 35°C (reversible propagation model)

$[LLA]_0$ (M)[a]	$[MBA]_0$ (mM)[a]	$[TBD]_0$ (mM)[a]	X_1 (%)[a]	X_5 (%)[a]	X_{10} (%)[a]	$[LLA]_{eq}$ (M)[b]	k_{obs} (s^{-1})[b]	$k_{obs}([LLA]_0 - [LLA]_{eq})$ ($M \cdot s^{-1}$)[c]
0.20	2.0	1.0	12.47	20.36	25.40	0.1531	0.6968	0.0327
0.22	2.2	1.1	14.42	25.33	28.94	0.1591	0.7029	0.0428
0.24	2.4	1.2	16.16	28.44	32.20	0.1658	0.7105	0.0527
0.26	2.6	1.3	15.56	30.77	35.82	0.1698	0.5506	0.0497
0.28	2.8	1.4	16.02	35.96	39.32	0.1700	0.5150	0.0567
0.30	3.0	1.5	18.13	41.53	42.64	0.1698	0.5567	0.0725
0.32	3.2	1.6	17.39	43.23	46.85	0.1678	0.4635	0.0705
0.34	3.4	1.7	18.37	48.06	50.95	0.1624	0.4572	0.0812
0.36	3.6	1.8	18.62	47.64	55.29	0.1580	0.3911	0.0790
0.38	3.8	1.9	18.46	49.86	60.00	0.1455	0.3408	0.0799
0.40	4.0	2.0	19.88	56.21	63.50	0.1383	0.3768	0.0986
0.42	4.2	2.1	22.33	59.65	65.62	0.1381	0.4187	0.1180
0.44	4.4	2.2	23.06	60.86	68.60	0.1322	0.4035	0.1242
0.46	4.6	2.3	22.09	63.79	72.52	0.1152	0.3653	0.1260
0.48	4.8	2.4	24.27	64.10	76.01	0.1071	0.3606	0.1345
0.50	5.0	2.5	26.17	66.66	81.01	0.0874	0.3523	0.1454
0.52	5.2	2.6	27.29	69.95	84.98	0.0693	0.3503	0.1579
0.54	5.4	2.7	28.35	71.67	86.29	0.0668	0.3634	0.1720
0.56	5.6	2.8	27.76	75.58	88.07	0.0541	0.3647	0.1845
0.58	5.8	2.9	30.05	76.82	88.76	0.0578	0.3954	0.2065
0.60	6.0	3.0	33.49	76.90	89.59	0.0649	0.4397	0.2353
0.62	6.2	3.1	34.36	79.91	91.12	0.0557	0.4534	0.2558
0.64	6.4	3.2	33.82	83.51	92.50	0.0415	0.4481	0.2682
0.66	6.6	3.3	34.62	84.64	93.38	0.0375	0.4566	0.2843
0.68	6.8	3.4	36.66	85.76	94.39	0.0355	0.4831	0.3113
0.70	7.0	3.5	39.32	87.95	95.46	0.0313	0.5243	0.3506
0.72	7.2	3.6	39.75	89.49	97.47	0.0177	0.5167	0.3629
0.74	7.4	3.7	39.59	89.99	99.49	0.0043	0.4965	0.3653
0.76	7.6	3.8	41.03	90.74	99.46	0.0063	0.5229	0.3941
0.78	7.8	3.9	42.89	92.09	98.22	0.0139	0.5701	0.4368
0.80	8.0	4.0	43.11	93.10	98.51	0.0097	0.5729	0.4528

[a] Data from Table S6, Zhang and Junkers, *Chem. Sci.* 2026, **17**, 4706-4714. [b] Apparent equilibrium monomer concentration $[LLA]_{eq}$ and first-order rate constant k_{obs} obtained by fitting $\hat{X} = \left(1 - \frac{[LLA]_{eq}}{[LLA]_0}\right)\left(1 - \exp\left(-k_{obs}t\right)\right)$ to conversion data. [c] Initial rate of monomer consumption predicted from $[LLA]_{eq}$ and k_{obs}.

Table S17. Selected literature data for $[LLA]_{eq}$ at 20°C

catalyst[a]	solvent	temperature[b]	ΔH (kJ/mol)	ΔS (J/mol/K)	$[LLA]_{eq}$ at 20°C (M)[c]	reference[d]
$Sn(Oct)_2$	dioxane	80-133°C	-22.9	-41.1	0.012	7
$Sn(Oct)_2$	bulk	180-220°C	-23.3	-22.0	0.001	8
DBU	dioxane	55-85°C	-15.1[e]	-23.1[e]	0.033	9

[a] $Sn(Oct)_2$: tin(II) 2-ethylhexanoate; DBU: 1,8-diazabicyclo(5.4.0)undec-7-ene. [b] range of temperatures used to calculate thermodynamic parameters. [c] $[LLA]_{eq} = \exp(\Delta H/RT - \Delta S/R)$. [d] numbers refer to references in main article. [e] calculated from data in reference 9, Supporting Table 3.

Table S18. Estimated equilibrium lactide concentrations, $[LLA]_{eq}$[a]

T (°C)	$[LLA]_{eq}$ (M)
0	0.0207
10	0.0262
20	0.0326
30	0.0400
35	0.0441

[a] calculated from data in reference 9, Supporting Table 3 (ΔH = -15.1 kJ/mol, ΔS= -23.1 J/mol/K). See Table S17, entry 3 for details of reaction conditions.

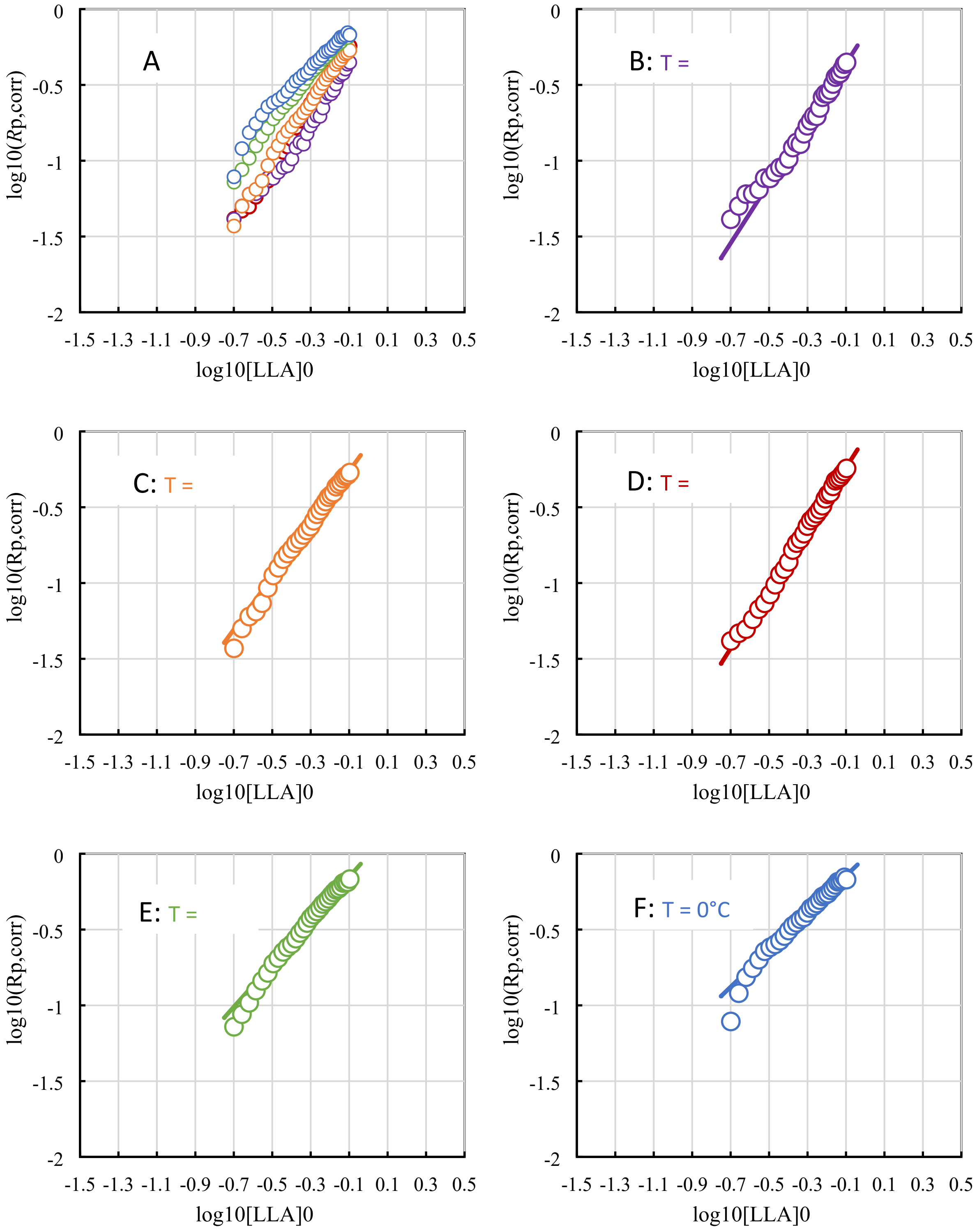

Figure S5. Log-log plots of depolymerization-corrected initial rate of lactide consumption vs initial lactide concentration for all monomer sweep experiments (A) and for individual temperatures showing overall reaction order (B-F). This is a depolymerization-corrected version of Figure S1, corrected using the equilibrium lactide concentrations shown in Table S18.

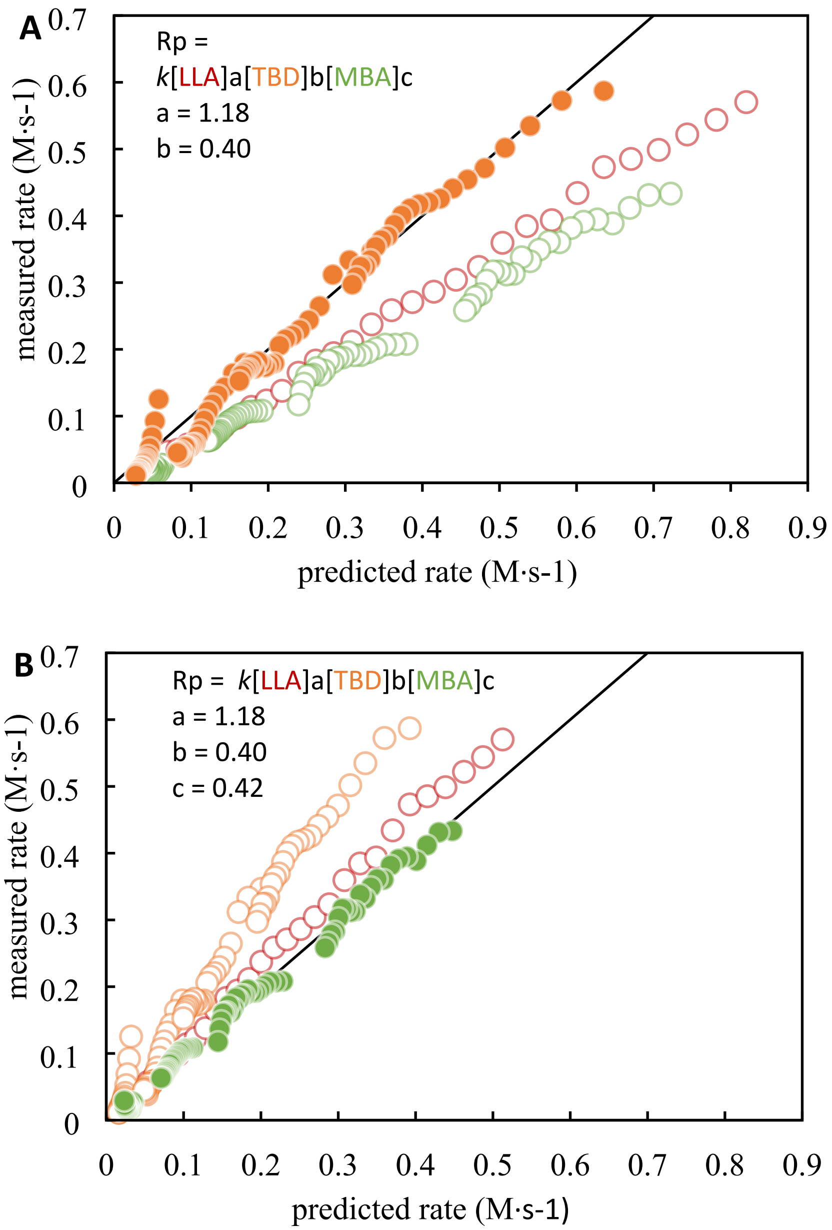

Figure S6. Comparison of predicted and measured depolymerization-corrected initial rates $(-d[LLA]/dt)_0 \times [LLA]_0/([LLA]_0-[LLA]_{eq})$ for monomer-to catalyst sweep (A) and degree of polymerization sweep (B) experiments. In each graph, the model is fit to the filled points, while the open circles represent the predictions of the model applied to the remaining experiments. In each panel, the exponent not determined by the corresponding dataset is obtained from the complementary dataset. Red: monomer concentration sweep (Tables S3-S7); orange: catalyst-to-monomer sweep (Tables S8-S10); green: DP sweep (Tables S11-S15). This is a depolymerization-corrected version of Figure 3, corrected using the equilibrium lactide concentrations shown in Table S18.

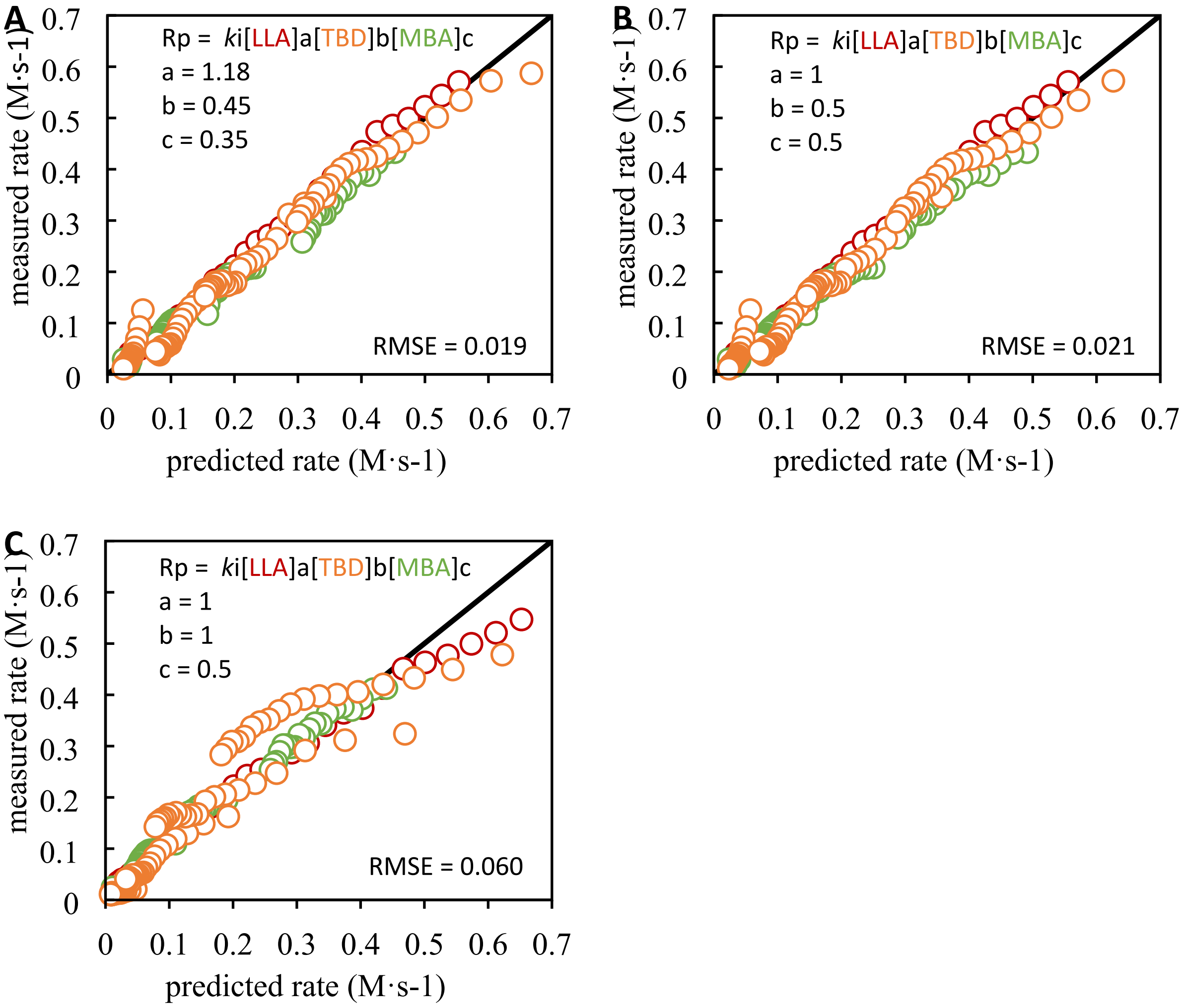

Figure S7. Comparison of predicted and measured depolymerization-corrected initial rates $(-d[LLA]/dt)_0 \times [LLA]_0/([LLA]_0-[LLA]_{eq})$ for all experiments, allowing k_i to vary depending on the experiment series (k_1: monomer-to-catalyst sweep; k_2: monomer concentration and DP sweep) (A) Empirical best fit from multiple weighted linear regression; (B) first-order in [LLA], half-order in [TBD] and [MBA]; (C) first-order in [LLA] and [TBD], half-order in [MBA]. Red: monomer concentration sweep (Tables S3-S7); orange: catalyst-to-monomer sweep (Tables S8-S10); green: DP sweep (Tables S11-S15). This is a depolymerization-corrected version of Figure 4, corrected using the equilibrium lactide concentrations shown in Table S18.

.
